# Demosponge diversity from North Sulawesi, with the description of six new species

**DOI:** 10.3897/zookeys.680.12135

**Published:** 2017-06-20

**Authors:** Barbara Calcinai, Azzurra Bastari, Giorgio Bavestrello, Marco Bertolino, Santiago Bueno Horcajadas, Maurizio Pansini, Daisy M. Makapedua, Carlo Cerrano

**Affiliations:** 1 Dipartimento di Scienze della Vita e dell’Ambiente, Università Politecnica delle Marche, Via Brecce Bianche, 60131, Ancona, UO Conisma, Italy; 2 Dipartimento di Scienze della Terra, dell’Ambiente e della Vita, Università degli Studi di Genova, Corso Europa, 26, 16132, Genova, UO Conisma, Italy; 3 Pharma Mar S.A.U. Av. de los Reyes, 1, 28770 Colmenar Viejo, Community of Madrid, Spain; 4 University of Sam Ratulangi, Jl. Kampus Unsrat 95115, Manado, Indonesia

**Keywords:** associations, diversity, Indonesia, new species, Porifera

## Abstract

Sponges are key components of the benthic assemblages and play an important functional role in many ecosystems, especially in coral reefs. The Indonesian coral reefs, located within the so-called “coral triangle”, are among the richest in the world. However, the knowledge of the diversity of sponges and several other marine taxa is far from being complete in the area. In spite of this great biodiversity, most of the information on Indonesian sponges is scattered in old and fragmented literature and comprehensive data about their diversity are still lacking. In this paper, we report the presence of 94 species recorded during different research campaigns mainly from the Marine Park of Bunaken, North Sulawesi. Six species are new for science and seven represent new records for the area. Several others are very poorly known species, sometimes recorded for the second time after their description. For most species, besides field data and detailed descriptions, pictures *in vivo* are included. Moreover, two new symbiotic sponge associations are described.

This work aims to increase the basic knowledge of Indonesian sponge diversity as a prerequisite for monitoring and conservation of this valuable taxon.

## Introduction

Baseline knowledge on species and assemblages is indispensable for monitoring the more and more frequent changes in biodiversity (Bell and Smith 2004). Sponges are often a key component of the benthic fauna for their abundance, dominance, wide pattern of interactions they develop (e.g. Cerrano et al. 2006, Wulff 2006, Wulff 2012, Bell 2008), longevity (Hogg et al. 2010) and role in the functioning of several ecosystems (Scheffers et al. 2010, de Goeij et al. 2013). Unfortunately, also due to the lack of taxonomic expertise, sponges are usually not considered in monitoring surveys and conservation programs (Bell and Smith 2004, Bell 2008).

The Indonesian archipelago, with its large number of islands (more than 17,000), hosts various and diversified habitats supporting high levels of diversity and endemism in marine life; this exceptional biodiversity is also the result of its geographic location and geological history (Tomascik et al. 1997). However, the impressive diversity of several marine taxa, such as sponges, corals, molluscs, ascidians etc., is still poorly known (Tomascik et al. 1997).

The knowledge on Indonesian sponges is mainly based on old expedition reports (such as Snellius II and Siboga expeditions) and on fragmented, recent studies including a few genera revisions (Hofman and van Soest 1995, de Voogd and van Soest 2002, Sim-Smith and Kelly 2011, Becking 2013) and new species descriptions (Azzini et al. 2008, de Weerdt and van Soest 2001, de Voogd 2003, de Voogd 2004, Calcinai et al. 2005a, Calcinai et al. 2006, Calcinai et al. 2007, de Voogd and van Soest 2007, de Voogd et al. 2008, Calcinai et al. 2013, Muricy 2011); for a more complete list, see also van Soest (1990). A few other papers concerning sponge ecology, distribution and symbiosis (Bavestrello et al. 2002, Bell and Smith 2004, Calcinai et al. 2004, Cerrano et al. 2006, de Voogd and Cleary 2008, de Voogd et al. 2009, Powell et al. 2014, Rossi et al. 2015) have been published.

In this paper, a list of 94 sponge species collected during several research expeditions conducted in this area is reported, and six new species are described from the North Sulawesi peninsula. Moreover, two new symbiotic associations are documented.

The aim of this study is to improve the knowledge on sponge diversity and distribution of North Sulawesi, a prerequisite for any study of monitoring and conservation of tropical coral reef assemblages.

## Materials and methods

**Figure 1.**
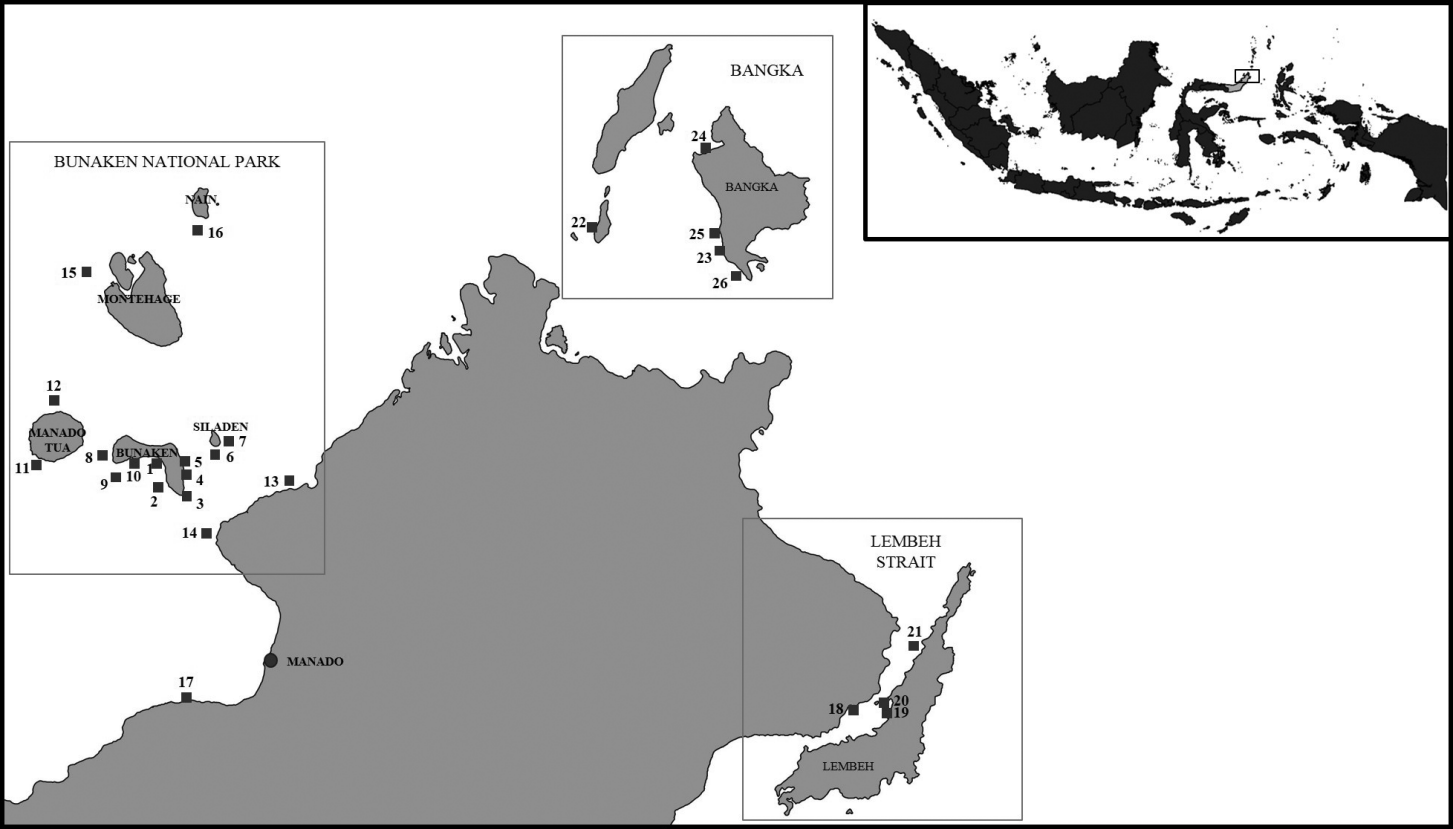
Locality map of North Sulawesi area showing the sponge collection sites. Black squares are the sampling sites. Key: **1** Liang **2** Lekuan II **3** Depan Kampung **4** Pangalisan **5** Timur **6** Siladen Jetty **7** Siladen Barat **8** Raymond’s Point **9** Fukui **10** Aluang Banua **11** Bualo **12** Tanjung Kopi **13** Tiwoho **14** Tanjung Pisok **15** Barracuda Point **16** Nain **17** Mapia Resort **18** Police Pier **19** Lembeh **20** Pintu Kolada **21** Angel’s window **22** Gangga Jetty **23** Bangka 2 **24** Bangka2 **25** Busa Bora **26** Yellow Coco.

The Bunaken Park is located in the northwest part of Sulawesi Island, Indonesia, in the coral triangle. It covers a total surface area of more than 89,000 hectares and includes five principal islands (Bunaken, Manado Tua, Mantehage, Nain and Siladen) (Fig. 1). Reefs can show different degrees of conservation (Fava et al. 2009) due to different anthropogenic impacts. The Lembeh Strait is a long, narrow, calm, and sheltered channel between the eastern coast of Sulawesi and the island of Lembeh that protects Bitung’s natural harbour. Bangka Island is an island of 4,778 hectares situated north of Manado, on the northeast tip of Sulawesi. Around the island, there are phanerogam meadows and mangrove forests as well as a reef with different steepness degrees (Calcinai et al. 2016).

The studied collection is the result of several expeditions performed in different years (August 1999, March 2000, May 2001, May 2002, September 2003, June 2004, January 2005) in the framework of bilateral agreements between Italy and Indonesia, focused on the exchange of researchers between the Italian Universities of Genoa and Polytechnic of Marche and the University of Sam Ratulangi (Manado, North Sulawesi). In May 2005, a further expedition in collaboration with the biopharmaceutical company Pharma Mar (http://www.pharmamar.com) was organised. In 2011, an expedition at Bangka Island in the frame of a joint project between Sam Ratulangi University and the Polytechnic University of Marche allowed to characterise the diversity of Porifera inside two small mangrove forests. Table 1 shows a list of all species and their distributions. In the Suppl. material 1, we included underwater photos of the species.

**Table 1.** List of the families and species of sponges collected during several research expeditions in the North Sulawesi peninsula, with sampling sites and depth ranges. Numbers in bold (1–26) match the sampling sites showed in Fig. 1. The samples, here indicated in bold, are showed in Suppl. material 1, Figure 11. The asterisk (*) marks the species considered as new records for the Indonesian area.

Family	Specie	Samples	Figure	Notes	1	2	3	4	5	6	7	8	9	10	11	12	13	14	15	16	17	18	19	20	21	22	23	24	25	26	Depth (m)
Plakinidae	*Plakortis lita* de Laubenfels, 1954	**PH33**, BU88	Figs 1–1	Short description and discussion in Chianese et al. 2012	-	√	-	√	-	-	-	-	-	-	-	-	-	-	-	-	-	-	-	-	-	-	-	-	-	-	20–24
Suberitidae	*Aaptos lobata* sp. n.	BU82, BU580, **PH1, PH27**, INDO079, INDO278, INDO339, INDO336	Fig. 2	This work	-	√	-	-	√	-	-	-	-	√	√	√	-	√	-	-	-	-	-	-	-	-	-	-	-	-	16.5–20
Halichondriidae	*Amorphinopsis excavans* Carter, 1887	MA1, **MA20**	Figs 11–2		-	-	-	-	-	-	-	-	-	-	-	-	-	-	-	-	-	-	-	-	-	-	√	√	-	-	0–1
Halichondriidae	*Ciocalypta tyleri* Bowerbank, 1873	**MA5**	Figs 11–3		-	-	-	-	-	-	-	-	-	-	-	-	-	-	-	-	-	-	-	-	-	-	√	-	-	-	0–1
Halichondriidae	*Topsentia halichondrioides* (Dendy, 1905)	**MA18**	Figs 11–4		-	-	-	-	-	-	-	-	-	-	-	-	-	-	-	-	-	-	-	-	-	-	-	√	-	-	0–1
Tethyidae	*Tethytimea tylota* (Hentschel, 1912)	BU98, BU289, BU533, BU545, **BU562**	Fig. 3	This work	-	√	-	-	-	-	-	√	-	-	√	-	-	-	-	-	-	-	-	-	-	-	-	-	-	-	5–20
Clionaidae	*Cliona albimarginata* Calcinai, Bavestrello & Cerrano, 2005	BU50, BU237		See picture in Calcinai et al. 2005a	-	-	-	-	-	-	-	-	-	-	-	-	-	-	√	-	-	-	-	-	-	-	-	-	-	-	7–25
Clionaidae	*Cliona favus* Calcinai, Bavestrello & Cerrano, 2005	BU11, BU54, BU473		See picture in Calcinai et al. 2005a	-	-	-	-	-	-	-	-	√	-	-	-	-	-	√	-	-	-	-	-	-	-	-	-	-	-	5–10
Clionaidae	*Cliona liangae* Calcinai, Bavestrello & Cerrano, 2005	MG1 bis		See picture in Calcinai et al. 2005a	√	-	-	-	-	-	-	-	-	-	-	-	-	-	-	-	-	-	-	-	-	-	-	-	-	-	Tidal zone
Clionaidae	*Cliona jullieni* Topsent, 1891	BU18, BU52, **BU58**, BU61, BU62, BU121, BU268	Fig. 11–5		-	√	-	-	-	-	-	-	√	√	-	-	-	-	√	√	-	-	-	-	-	-	-	-	-	-	3–17
Clionaidae	*Cliona mucronata* Sollas, 1878	**BU16bis**, BU79, BU501,	Fig. 11–6	*In situ* photo not available	-	√	-	-	-	-	-	-	-	√	-	-	-	-	√	-	-	-	-	-	-	-	-	-	-	-	5–21
Clionaidae	*Cliona orientalis* Thiele, 1900	BU73, **BU319**, BU119	Fig. 11–7		-	-	√	-	-	-	-	-	-	-	√	-	-	-	-	-	-	-	-	-	-	-	-	-	-	-	5–25
Clionaidae	*Cliona schmidtii* (Ridley, 1881)	BU56, **BU58**, BU75, BU83bis, BU255, BU264, BU322	Fig. 11–8		-	√	√	-	-	-	-	√	-	-	-	-	-	-	-	√	-	-	-	-	-	-	-	-	-	-	10–21
Clionaidae	*Cliona utricularis* Bavestrello & Cerrano, 2005	**BU13**, BU31, BU60, BU115, BU136, BU145, BU474, BU482, BU500, BU505	Fig. 11–9	*In situ* photo not available	√	-	-	-	-	√	-	-	√	-	-	-	-	-	√	√	-	-	√	-	-	-	-	-	-	-	5–16
Clionaidae	*Cliothosa aurivilli* (Lindgren, 1897)	MG1, **BU72**	Fig. 11–10	*In situ* photo not available	-	-	√	√	-	-	-	-	-	-	-	-	-	-	-	-	-	-	-	-	-	-	-	-	-	-	0–1
Clionaidae	*Cliothosa hancocki* (Topsent, 1888)	**BU17**, BU120	Fig. 1–11	*In situ* photo not available	-	-	-	-	-	-	-	-	√	√	-	-	-	-	-	-	-	-	-	-	-	-	-	-	-	-	5
Clionaidae	*Pione carpenteri* (Hancock, 1867)	**BU74**	Fig. 1–12	*In situ* photo not available	-	√	-	-	-	-	-	-	-	-	-	-	-	-	-	-	-	-	-	-	-	-	-	-	-	-	9
Clionaidae	*Spheciospongia solida* (Ridley & Dendy, 1886)	BU45, BU228, BU576, **PH24**	Fig. 1–13		-	-	√	√	-	-	-	-	-	√	-	-	-	-	-	-	-	√	-	-	-	-	-	-	-	-	10–25
Clionaidae	*Spheciospongia vagabunda* (Ridley, 1884)	BU44, **BU64**, BU296	Fig. 1–14	*In situ* photo not available	-	-	√	-	-	-	-	-	-	-	-	-	-	√	-	-	-	-	-	-	-	-	-	-	-	-	5–20
Spirastrellidae	*Spirastrella pachyspira* Lévi, 1958 *	**BU78**	Fig. 1-2	See Suppl. material 2	-	√	-	-	-	-	-	-	-	-	-	-	-	-	-	-	-	-	-	-	-	-	-	-	-	-	25
Acarnidae	*Zyzzya fuliginosa* (Carter, 1879)	**BU53**, BU78, BU260, BU265, BU266	Fig. 1-15		-	√	-	-	-	-	-	-	-	-	-	-	-	-	√	-	-	-	-	-	-	-	-	-	-	-	10–30
Chondropsidae	*Chondropsis subtilis* Calcinai, Bavestrello, Bertolino, Pica, Wagner & Cerrano, 2013	Bugor504	See picture in Calcinai et al. 2013		-	-	-	-	-	√	-	-	-	-	√	-	-	-	-	-	-	-	-	-	-	-	-	-	-	-	10–15
Crambeidae	*Monanchora enigmatica* (Burton & Rao, 1932)	**PH45**, Bugor410	Fig. 1-16		-	-	-	-	√	√	-	-	-	-	-	-	-	-	-	-	-	-	-	-	-	-	-	-	-	-	20–27
Desmacididae	*Desmapsamma vervoorti* van Soest, 1998	**BU222**, BU410, BU411, Bugor410, Carramba1, Carramba2, Carramba6, Carramba8, BA4	Fig. 1-17		-	-	-	-	-	√	-	-	-	-	-	-	-	-	-	-	-	-	√	√	-	-	-	-	-	-	1–27
Hymedesmiidae	Hymedesmia (Hymedesmia) spinata Calcinai, Bavestrello, Bertolino, Pica, Wagner & Cerrano, 2013	Bugor513, Bugor311, Bugor309, Bugor410bis2	See picture in Calcinai et al. 2013		-	-	-	-	-	√	√	-	-	-	-	-	-	-	-	-	-	-	-	-	-	-	-	-	-	-	23–26
Hymedesmiidae	Hymedesmia (Stylopus) perlucida Calcinai, Bavestrello, Bertolino, Pica, Wagner & Cerrano, 2013	Bugor511	See picture in Calcinai et al., 2013		-	-	-	-	-	-	-	-	-	-	-	-	-	-	-	√	-	-	-	-	-	-	-	-	-	-	17
Isodictyidae	*Coelocarteria agglomerans* Azzini, Calcinai & Pansini, 2007	BU15, BU37, BU38, BU48, BU132, BU219, BU329, BU616, BU644 **PH51**	Fig. 1-18		-	-	-	-	-	√	-	√	-	√	-	-	√	-	-	-	-	-	-	-	√	-	-	-	-	-	20–38
Microcionidae	Clathria (Thalysias) cervicornis (Thiele, 1903)	**PH48**	Fig. 1-19		-	-	-	-	√	-	-	-	-	-	-	-	-	-	-	-	-	-	-	-	-	-	-	-	-	-	20
Microcionidae	Clathria (Thalysias) mutabilis (Topsent, 1897)	**PH19**	Fig. 1-20		-	-	-	-	√	-	-	-	-	-	-	-	-	-	-	-	-	-	-	-	-	-	-	-	-	-	20
Mycalidae	Mycale (Aegogropila) furcata Calcinai, Bavestrello, Bertolino, Pica, Wagner & Cerrano, 2013	Bugor307, Bugor332	See picture in Calcinai et al. 2013		-	-	-	-	-	√	√	-	-	-	-	-	-	-	-	-	-	-	-	-	-	-	-	-	-	-	Depth not stated
Mycalidae	Mycale (Mycale) corallina Calcinai, Cerrano & Bavestrello, 2016	BU485, BU534, BU449	See picture in Calcinai et al. 2006	See Suppl. material 2	-	-	-	-	-	√	-	-	-	-	√	-	-	-	-	-	-	-	-	-	-	-	-	-	-	-	17–20
Podospongiidae	*Podospongia colini* Sim-Smith & Kelly, 2011		Fig. 1-21	Not available data; *in situ* photo not available	-	-	-	-	-	-	-	-	-	-	-	-	-	-	-	-	-	-	-	-	-	-	-	-	-	-	Depth not stated
Tedaniidae	Tedania (Tedania) brevispiculata Thiele, 1903	**MA21**	Fig. 1-22	*In situ* photo not available	-	-	-	-	-	-	-	-	-	-	-	-	-	-	-	-	-	-	-	-	-	-	-	√	-	-	1
Tedaniidae	Tedania (Tedania) coralliophila Thiele, 1903	**BU582**	Fig. 1-23		-	-	-	-	-	-	-	-	-	√	-	-	-	-	-	-	-	-	-	-	-	-	-	-	-	-	11
Tedaniidae	Tedania (Tedania) dirhaphis Hentschel, 1912	**PH52**	Fig. 1-24		-	-	-	-	-	-	-	-	-	-	-	-	√	-	-	-	-	-	-	-	-	-	-	-	-	-	20
Agelasidae	*Agelas ceylonica* Dendy, 1905	BU1, BU3, **PH54**	Fig. 1-25		-	-	-	-	-	-	-	-	-	√	-	-	√	-	-	-	-	-	-	-	-	-	-	-	-	-	20–30
Agelasidae	*Agelas mauritiana* (Carter, 1883)	BU234, **BU570**	Fig. 1-26		-	-	-	-	-	-	-	-	-	√	-	-	-	-	-	-	-	-	-	-	-	-	-	-	-	-	23–35
Agelasidae	*Agelas nakamurai* Hoshino, 1985	BU583, **PH38**	Fig. 1–27		-	-	-	-	√	-	-	-	-	√	-	-	-	-	-	-	-	-	-	-	-	-	-	-	-	-	12–20
Tetillidae	*Cinachyrella australiensis* (Carter, 1886)	**BU233**, BU308, BU316	Fig. 1–28		-	-	-	-	-	-	-	-	-	√	√	-	-	-	-	-	-	-	-	-	-	-	-	-	-	-	5–13
Tetillidae	*Tetilla ridleyi* Sollas, 1888	**MA17**	Fig. 1–29	*In situ* photo not available	-	-	-	-	-	-	-	-	-	-	-	-	-	-	-	-	-	-	-	-	-	-	√	-	-	-	1
Ancorinidae	Dercitus (Stoeba) bangkae (Calcinai, Bastari, Makapedua, Cerrano, 2016)	**MA16**	Fig. 1–30	*In situ* photo not available	-	-	-	-	-	-	-	-	-	-	-	-	-	-	-	-	-	-	-	-	-	-	√	-	-	-	1
Ancorinidae	*Rhabdastrella distincta* (Thiele, 1900)	**BU560**, **BU575**	Fig. 5, Fig. 6	This work	-	-	-	-	-	-	-	-	-	√	√	-	-	-	-	-	-	-	-	-	-	-	-	-	-	-	Max. depth 30
Ancorinidae	*Rhabdastrella globostellata* (Carter, 1883)	BU288, **BU593**, PH29	Fig. 1–31		-	√	-	-	√	-	-	-	-	-	-	-	-	-	-	-	-	-	-	-	-	-	-	-	-	-	20–23
Ancorinidae	*Stelletta clavosa* Ridley, 1884	**BU543**	Fig. 1–32	See Suppl. material 2	-	√	-	-	-	-	-	√	√	-	-	-	-	-	-	-	-	-	-	-	-	-	-	-	-	-	5–30
Ancorinidae	*Stelletta tethytimeata* sp. n.	BU98, BU289, **BU533**, BU545, BU562	Fig. 4	This work	-	√	-	-	-	-	-	√	-	-	√	-	-	-	-	-	-	-	-	-	-	-	-	-	-	-	5–20
Geodiidae	*Melophlus sarasinorum* Thiele, 1899	**PH56**	Fig. 1–33	See Suppl. material 2	-	-	-	-	-	-	-	-	-	-	-	-	√	-	-	-	-	-	-	-	-	-	-	-	-	-	20
Theonellidae	*Theonella cylindrica* Wilson, 1925	**BU568**	Fig. 1–34		-	-	-	-	-	-	-	-	-	√	-	-	-	-	-	-	-	-	-	-	-	-	-	-	-	-	40
Theonellidae	*Theonella mirabilis* (de Laubenfels, 1954)	**BU585**	Fig. 1–35	See Suppl. material 2	-	-	-	-	-	-	-	-	-	√	-	-	-	-	-	-	-	-	-	-	-	-	-	-	-	-	11
Theonellidae	*Theonella swinhoei* Gray, 1868	BU96, PH12, **PH60**	Fig. 1–36		-	√	-	-	√	-	-	-	-	-	-	-	√	-	-	-	-	-	-	-	-	-	-	-	-	-	20–25
Thoosidae	*Thoosa letellieri* Topsent, 1891	MTR	Fig. 15	See Suppl. material 2	-	-	-	√	-	-	-	-	-	-	-	-	-	-	-	-	-	-	-	-	-	-	-	-	-	-	Depth not stated
Biemnidae	*Biemna fortis* (Topsent, 1897)	**BU143**, MA11	Fig. 1–37	*In situ* photo not available	-	-	-	-	-	-	-	-	-	-	-	-	-	-	-	-	-	-	√	-	-	-	√	-	-	-	1–30
Dictyonellidae	*Acanthella cavernosa* Dendy, 1922	**PH53**	Fig. 1–38		-	-	-	-	-	-	-	-	-	-	-	-	√	-	-	-	-	-	-	-	-	-	-	-	-	-	20
Dictyonellidae	*Phakettia ridley* (Dendy, 1887) *	**BU578**	Fig. 1–39		-	-	-	-	-	-	-	-	-	√	-	-	-	-	-	-	-	-	-	-	-	-	-	-	-	-	20
Scopalinidae	*Stylissa carteri* (Dendy, 1889)	**PH59**	Fig. 1–40		-	-	-	-	-	-	-	-	-	-	-	-	√	-	-	-	-	-	-	-	-	-	-	-	-	-	20
Scopalinidae	*Stylissa massa* (Carter, 1887)	**PH57**	Fig. 1–41		-	-	-	-	-	-	-	-	-	-	-	-	√	-	-	-	-	-	-	-	-	-	-	-	-	-	25
Callyspongiidae	Callyspongia (Cladochalina) aerizusa Desqueyroux-Faúndez, 1984	BU242, **BU577**, PH15	Fig. 1–42		-	-	-	√	-	-	-	-	-	√	-	-	-	-	-	√	-	-	-	-	-	-	-	-	-	-	7–20
Callyspongiidae	Callyspongia (Cladochalina) fibrosa (Ridley & Dendy, 1886)	**PH2**	Fig. 1–43	*In situ* photo not available	-	-	-	-	√	-	-	-	-	-	-	-	-	-	-	-	-	-	-	-	-	-	-	-	-	-	20
Chalinidae	*Chalinula nematifera* (de Laubenfels, 1954)		Fig. 1–44		-	-	-	-	-	-	-	-	-	-	-	-	-	-	-	-	-	-	-	-	-	√	-	-	√	-	
Chalinidae	*Cladocroce burapha* Putchakarn et al. 2004	MA6, MA19a, **MA19c**, MA19e	Fig. 1–45		-	-	-	-	-	-	-	-	-	-	-	-	-	-	-	-	-	-	-	-	-	-	√	-	-	-	0–1
Chalinidae	Haliclona (Reniera) fascigera (Hentschel, 1912)	**PH8**	Fig. 1–46		-	-	-	-	√	-	-	-	-	-	-	-	-	-	-	-	-	-	-	-	-	-	-	-	-	-	20
Chalinidae	Haliclona (Halichoclona) centrangulata (Sollas, 1902)	**MA4**	Fig. 1–47		-	-	-	-	-	-	-	-	-	-	-	-	-	-	-	-	-	-	-	-	-	-	√	-	-	-	0–1
Niphatidae	*Amphimedon anastomosa* sp. n.	PH58	Fig. 7	This work	-	-	-	-	-	-	-	-	-	-	-	-	√	-	-	-	-	-	-	-	-	-	-	-	-	-	20
Niphatidae	Amphimedon cf. sulcata Fromont, 1983	**BU560**, **BU560-a1**, **BU575**	Fig. 5, Fig. 6		-	-	-	-	-	-	-	-	-	√	√	-	-	-	-	-	-	-	-	-	-	-	-	-	-	-	Max. depth 30
Niphatidae	*Dasychalina fragilis*	**BA8**	Fig. 1–48	Data not available	-	-	-	-	-	-	-	-	-	-	-	-	-	-	-	-	-	-	-	-	-	-	-	-	-	-	Depth not stated
Niphatidae	*Gelliodes fibulata* (Carter, 1881)	**PH20**	Fig. 1–49	*In situ* photo not available	-	-	-	-	√	-	-	-	-	-	-	-	-	-	-	-	-	-	-	-	-	-	-	-	-	-	20
Niphatidae	*Gelliodes hamata* Thiele, 1903	Bugor Onong	See picture in Calcinai et al. 2013		-	-	-	-	-	√	-	-	-	-	-	-	-	-	-	-	-	-	-	-	-	-	-	-	-	-	28
Niphatidae	*Niphates olemda* (de Laubenfels, 1954)	BU581, BU587, **BU591**, BU597, PH7	Fig. 1–50		-	-	-	-	√	-	-	-	-	√	-	-	-	-	-	-	-	-	-	-	-	-	-	-	-	-	8–25
Niphatidae	*Niphates laminaris* sp. n.	PH47	Fig. 8	This work	-	-	-	-	-	-	-	-	-	-	-	-	√	-	-	-	-	-	-	-	-	-	-	-	-	-	20
Petrosiidae	*Acanthostrongylophora ingens* (Thiele, 1899)	BU4, BU35, BU133, BU134, BU297, BU298, BU302, BU320, BU323, BU544, **PH11**, PH16	Fig. 1–51	See Suppl. material 2	-	√	-	-	√	√	-	√	-	√	-	-	-	√	-	-	-	-	-	-	-	-	-	-	-	-	8–30
Petrosiidae	*Neopetrosia seriata* (Hentschel, 1912)	BU76, BU83, **BU324**, BU508, BU513	Fig. 1–52	*In situ* photo not available	-	√	-	-	-	-	-	√	-	-	-	-	-	-	-	-	-	-	-	-	-	-	-	-	-	-	30–65
Petrosiidae	*Neopetrosia similis* (Ridley & Dendy, 1886) *	BU122	Fig. 1–53		-	-	-	-	-	-	-	√	-	-	-	-	-	-	-	-	-	-	-	-	-	-	-	-	-	-	Depth not stated
Petrosiidae	Petrosia (Petrosia) hoeksemai de Voogd & van Soest, 2002	BU518, BU520, **PH14**	Fig. 1–54		-	-	-	√	-	-	-	√	-	-	-	-	-	-	-	-	-	-	-	-	-	-	-	-	-	-	3–20
Petrosiidae	Petrosia (Petrosia) nigricans Lindgren, 1897	BU1, BU92, BU93, BU232, BU284, BU286, BU299, BU344, BU512, BU517, BU531, **BU571**, BU572, PH10	Fig. 1–55		-	√	-	-	√	-	-	√	-	√	-	-	-	√	-	-	√	-	-	-	-	-	-	-	-	-	4–32
Petrosiidae	Petrosia (Petrosia) plana Wilson, 1925	BU97, BU285, BU509, BU515, **BU565**, BU410	Fig. 1–56		-	√	-	-	-	-	-	√	-	√	-	-	-	-	-	-	-	-	-	-	-	-	-	-	-	-	7–43
Petrosiidae	Petrosia (Petrosia) seychellensis Dendy, 1922 *	**BU595**	Fig. 1–57		-	-	-	-	√	-	-	-	-	-	-	-	-	-	-	-	-	-	-	-	-	-	-	-	-	-	20
Petrosiidae	Petrosia (Strongylophora) corticata (Wilson, 1925)	**BU102**, BU277	Fig. 1–58		-	-	-	-	-	-	-	√	-	-	-	-	-	-	-	-	-	-	-	-	-	-	-	-	-	-	25–30
Petrosiidae	Petrosia (Strongylophora) durissima (Dendy, 1905) *	**PH39**	Fig. 1–59	*In situ* photo not available	-	-	-	√	-	-	-	-	-	-	-	-	-	-	-	-	-	-	-	-	-	-	-	-	-	-	20
Petrosiidae	Petrosia (Strongylophora) strongylata Thiele, 1903	**BU516**	Fig. 1–60	*In situ* photo not available	-	-	-	-	-	-	-	√	-	-	-	-	-	-	-	-	-	-	-	-	-	-	-	-	-	-	Depth not stated
Petrosiidae	*Xestospongia testudinaria* (Lamarck, 1815)	BU250, **BU510**	Fig. 1–61	*In situ* photo not available	-	-	√	-	-	-	-	√	-	-	-	-	-	-	-	-	-	-	-	-	-	-	-	-	-	-	Depth not stated
Phloeodictyidae	*Siphonodictyon maldiviensis* (Calcinai, Cerrano, Sarà & Bavestrello, 2000)	BU16, BU200, BU342	See picture in Calcinai et al., 2007		-	-	-	-	-	√	-	-	-	√	√	-	-	-	-	-	√	-	-	-	-	-	-	-	-	-	1–40
Phloeodictyidae	*Siphonodictyon microterebrans* (Calcinai, Cerrano & Bavestrello, 2007)	BU51, BU125, BU261, BU492, BU493, BU496b	See picture in Calcinai et al., 2007		-	√	-	-	-	√	-	√	-	-	√	-	-	-	√	-	-	-	-	-	-	-	-	-	-	-	5–30
Phloeodictyidae	*Siphonodictyon mucosum* Bergquist, 1965	BU19, **BU20**, BU450, BU484	Fig. 1–62	White arrows	-	-	-	-	-	√	-	-	√	-	√	-	-	-	-	-	-	-	-	-	-	-	-	-	-	-	6–40
Phloeodictyidae	*Oceanapia amboinensis* Topsent, 1897	BU25, BU65, BU147, BU257, BU321	See picture in Bavestrello et al., 2002	The aquiferous system of this species was described in Bavestrello et al., 2002	√	√	√	-	-	-	-	-	-	-	-	-	-	-	-	-	-	-	√	-	-	-	-	-	-	-	0–1
Phloeodictyidae	*Oceanapia fistulosa* (Bowerbank, 1873)	BU6, BU36, BU101, BU103, BU128, BU130, BU274, BU280, BU341, **PH43**, PH50, Bugor514	Fig. 1–63	The aquiferous system of this species was described in Bavestrello et al., 2002	-	-	-	√	-	√	-	√	-	√	-	-	√	-	-	-	√	-	-	-	-	-	-	-	-	-	20–42
Phloeodictyidae	*Oceanapia pedunculata* (Ridley & Dendy, 1886)	BU64bis		Photos not available	-	-	√	-	-	-	-	-	-	-	-	-	-	-	-	-	-	-	-	-	-	-	-	-	-	-	Depth not stated
Phloeodictyidae	*Oceanapia seychellensis* (Dendy, 1922) *	**BU300**, BU290, BU328	Fig. 1–64	*In situ* photo not available	-	-	-	-	-	-	-	√	-	-	-	-	-	√	-	-	-	-	-	-	-	-	-	-	-	-	20–44
Phloeodictyidae	*Oceanapia toxophila* Dendy, 1922 *	BU276, **BU314**	Fig. 1–65	*In situ* photo not available	-	-	-	-	-	-	-	√	-	-	√	-	-	-	-	-	-	-	-	-	-	-	-	-	-	-	13–30
Irciniidae	*Ircinia colossa* sp. n.	BU590, PH44, **BKA 25**, INDO431	Fig. 10	This work	-	-	-	-	√	√	-	-	-	-	-	-	-	-	-	-	-	-	-	-	-	-	-	-	-	√	20–25
Irciniidae	*Psammocinia alba* sp. n.	PH41	Fig. 9	This work	-	-	-	-	√	-	-	-	-	-	-	-	-	-	-	-	-	-	-	-	-	-	-	-	-	-	20
Spongiidae	Spongia (Spongia) ceylonensis Dendy, 1905	**BU589**	Fig. 1–66		-	-	-	-	-	-	-	-	-	√	-	-	-	-	-	-	-	-	-	-	-	-	-	-	-	-	3.5
Thorectidae	*Hyrtios communis* (Carter, 1885)	**MA12**	Fig. 1–67		-	-	-	-	-	-	-	-	-	-	-	-	-	-	-	-	-	-	-	-	-	-	√	-	-	-	1
Thorectidae	*Hyrtios reticulatus* (Thiele, 1899)	**PH36**	Fig. 1–68		-	-	-	-	√	-	-	-	-	-	-	-	-	-	-	-	-	-	-	-	-	-	-	-	-	-	20
Thorectidae	*Phyllospongia papyracea* (Esper, 1794)	M3, M4, **PH6**, BA3	Fig. 1–69		-	-	-	-	√	-	-	-	-	-	-	-	-	-	-	-	-	-	-	-	-	-	-	-	-	-	20
Thorectidae	*Carteriospongia foliascens* (Pallas, 1766)	**BU112**, BU343	Fig. 1–70		-	-	-	-	-	-	-	√	-	-	-	-	-	-	-	-	√	-	-	-	-	-	-	-	-	-	5

Spicule preparations, for optical and scanning electron microscopy (SEM), were made according to Rützler (1974). Spicule dimensions were obtained by measuring 30 spicules per type. Maximal, minimal, and average sizes, ± standard deviation (length and width) are given. The skeletal architecture, under light and scanning electron microscope (SEM), was studied on hand-cut sections of sponge portions, following Hooper (2000). The SEM analysis was conducted using a Philips XL 20 SEM.

Histological sections were prepared from fragments of sponges fixed *in situ* in buffered 2.5% glutaraldehyde in artificial sea water, dehydrated in graded ethanol series, desilicified in 4% hydrofluoric acid, decalcified in 4% hydrochloride acid and embedded in Technovit 8100 (Kulzer). Other fragments were routinely paraffin-embedded and sectioned to obtain preparations of the associated sponges.

Comparative type material of *Acanthostrongylophora ingens* (Thiele, 1899) was kindly provided by The Naturhistorisches Museum at Basel (**NMB**) (Switzerland). Type material is deposited at the Museo di Storia Naturale di Genova Giacomo Doria (**MSNG**), Italy.

## Results

A total of 94 demosponge species belonging to 33 families is documented and identified; these species are listed in Table 1; seven of these are new records for the area (Table 1). Six new species were discovered and are herein described.

Seven species (*Tethytimea tylota* (Hentschel, 1912), *Rhabdastrella distincta* (Thiele, 1900), *Thoosa letellieri* Topsent, 1891, *Theonella mirabilis* (de Laubenfels, 1954), Tedania (Tedania) coralliophila Thiele, 1903, *Podospongia colini* Sim-Smith and Kelly, 2011 and Amphimedon cf. sulcata Fromont, 1993) were recorded for the first time since their original description; for those involved in symbiotic relationships (*T. tylota*, *R. distincta*, and A. cf. sulcata), extensive morphological and ecological remarks are added, while the others are otherwise briefly described in the Suppl. material 2. Additional taxonomic notes and pictures are added for *Acanthostrongylophora ingens* Thiele, 1889, *Spirastrella pachyspira* Lévi, 1958 and Mycale (Mycale) vansoesti *sensu* Calcinai, Cerrano, Totti, Romagnoli & Bavestrello, 2006. In vivo pictures of the listed species are given in Suppl. material 1.

## Taxonomy

### Class Demospongiae

#### Subclass Heteroscleromorpha

##### Order Suberitida Morrow & Cárdenas, 2015

###### Family Suberitidae

####### Genus *Aaptos* Gray, 1867

######## Aaptos lobata

Taxon classificationAnimaliaSuberitidaSuberitidae

Calcinai, Bastari, Bertolino & Pansini sp. n.

http://zoobank.org/A771C968-0DB7-406C-A3A8-9B56BE236ABF

Figure 2

######### Material examined.

Holotype: MSNG 60134, PH-1, 13/01/2005, Timur (Bunaken Island), about 20 m depth. Paratype: MSNG 60135, PH-27, 13/01/2005, same locality as holotype, about 20 m depth.

######### Other material.

BU-82, 22/03/2000, Lekuan II (Bunaken Island), 20 m depth. BU-580, 27/06/2004, Alung Banua (Bunaken Island), 16 m depth. INDO-079, 08/05/2005, Tanjung Kopi (Manado Tua), unknown depth, N01°39'07.4"; E124°41'58.8". INDO-278, 11/05/2005, Tansung Pisok (Manado), unknown depth, N01°34'31.2"; N01°34'31.2". INDO-336, 12/05/2005, Bualo (Manado), unknown depth, N01°37'00.7"; E124°41'21.9". INDO-339, 12/05/2005, Bualo (Manado), unknown depth, N01°37'00.7"; E124°41'21.9".

######### Diagnosis.

Cushion-shaped, sub-spherical sponge; yellow, brown or dark orange. Strongyloxeas, styles and subtylostyles not separable in size categories, forming ascending tracts protruding through the sponge surface.

######### Description.

**Figure 2.**
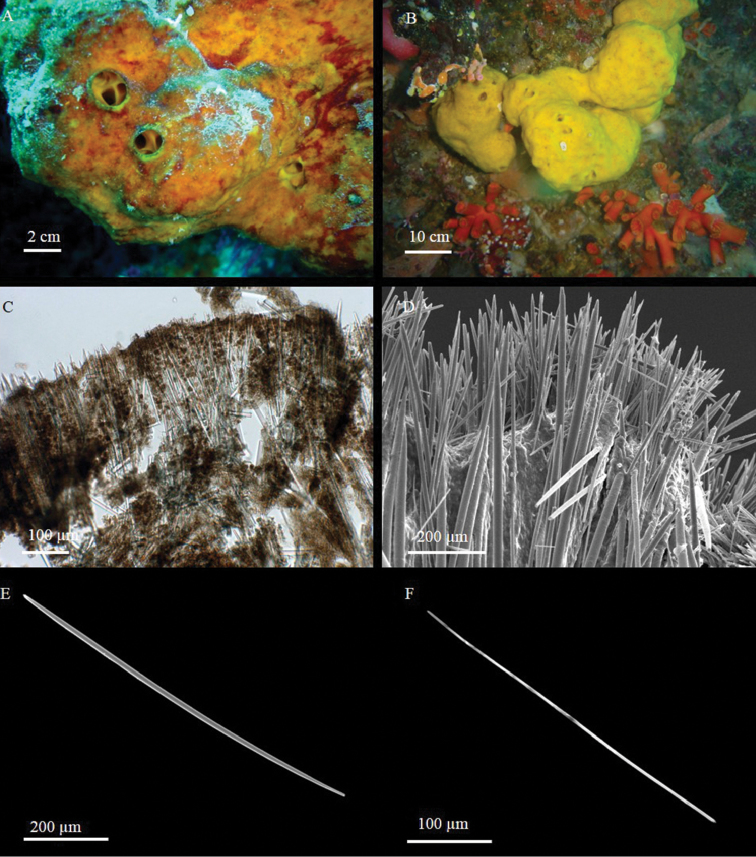
*Aaptos lobata* sp. n. **A, B** specimens *in situ*: **A** holotype **B** paratype **C** skeleton organisation (transverse section) **D** peripherical part of the skeleton **E** large strongyloxea **F** thin style.

The sponge is massive, sub-spherical or lobate (Fig. 2A, B). The holotype (Fig. 2A) is a fragment about 1.5 cm long and 1 cm thick, sampled from a large globular specimen; the paratype is a small portion, approximately 2.5 cm long and 1 cm thick, of a large cushion-shaped specimen approximately 60 cm across. The paratype (Fig. 2B) shows a sort of lobate organisation, with roundish parts connected by bottleneck narrowings. The colour in life is yellow, varying between orange and brown according to light exposure; it is not uniform, but presents dark red spots or stripes (Fig. 2A, B). The sponge is always yellow inside. Alcohol-preserved specimens are dark green-brown. The sponge surface is smooth, but microscopically hispid. Ostia, grouped in distinct areas on the sponge surface, have such a large diameter that they are visible to the naked eye. Oscula are flush, more or less circular, with a very low rim. Converging exhalant canals are visible in their lumen (Fig. 2A). Consistency is hard when preserved.

**Skeleton.** The choanosomal skeleton is radiate, regular in the outer part of the sponge and more irregular in the deeper part. Due to high spicule density, spicule tracts are not easily detectable (Fig. 2C, D). In the ectosome, the smallest styles are arranged in palisade and do not form brushes, whereas the spicules of intermediate size are concentrated in the sub-ectosomal layer and protrude through the surface with their tips (Fig. 2C, D). Abundant spheroulous cells, approximately 12 µm in diameter, are detectable in the choanosome.

**Spicules.** Three size categories of megascleres, partially overlapping at the extremities of their size-frequency distributions. The larger spicules are straight strongyloxeas with acerate or slightly stepped tips (Fig. 2E) and often evident axial canal. Intermediate and small megascleres, straight or slightly curved, vary in shape from strongyloxeas to subtylostyles to thin styles (Fig. 2F). The measurements are given in Table 2.

**Table 2.** *Aaptos* species distributed in the tropical Indo-Pacific and adjacent areas.

Species	Shape and surface	Colour	Consistence	Skeleton	Spicules (µm)
*A. ciliata* (Wilson, 1925)	Massive, lobate; surface conulose and hispid	Whitish brown	-	Collagenous ectosome 0,5 mm thick, with cavities Choanosome dense with ill-defined spicule tracts	Styles 1400–2000 × 20–36 Ectosomal styles 1100–1300 × 4
A. conferta Kelly-Borges & Bergquist, 1994	Thickly encrusting, lobate; surface smooth or micro-hispid	Jet black outside, mustard yellow inside	Just compressible	Stout megasclere tracts with interstitial spicules	Strongyloxeas 662–1813 × 13–29 2 categories of styles Oxeas 156–537 × 3–8
A. globosa Kelly-Borges & Bergquist, 1994	Spherical; surface smooth	Deep red brown outside, mustard yellow inside	Incompressible	Tracts of primary megascleres radiating at the surface; superficial palisade not piercing the sponge surface	Strongyloxeas I 980–2401 × 18–33 Strongyloxeas II 332–1029 × 8–16 Tylostyles 104–198 × 4–5 Subtylostyles 208–458 × 5–8
*A. horrida* (Carter, 1886)	Massive elongate; surface even and villous	Grey	Very compact	Very compact	2 size categories of fusiform, acerate spicules
*A. laxosuberites* (Sollas, 1902)	Encrusting; surface slightly hispid	Whitish, in spirit	-	Ascending and diverging tracts of megascleres Ectosomal skeleton of small styles	Strongyloxeas I 750–1120 × 26–40 II 250 × 4 Tylostyles 700 × 20
*A. niger* Hoshino, 1981	Massive, embedding extraneous material; surface minutely hispid	Black	Incompressible	Ectosome with small styles; radiate architecture and confused spicules in the choanosome	Strongyloxeas I 540–1310 × 18–46 II 170–270 × 5–10
*A. nuda* (Kirkpatrick, 1903)	Massive; surface finely papillate	Pale brown outside, interior lighter (in spirit)	Rather hard	Ill-defined bundles of oxeas radiating towards the surface	Oxeas 1700 × 45
*A. rosacea* Kelly-Borges & Bergquist, 1994	Spherical to semi spherical; surface smooth and faintly hispid	Oxide red outside and golden yellow inside	Incompressible	Choanosomal tracts of megascleres branching at the surface and forming tufts Superficial palisade of tylostyles and subtylostyles	Strongyloxeas 735–2009 × 10–23 Styles 367–1102 × 5–12 Tylostyles 94–218 × 3–8 Subtylostyles 198–447 × 4–13
*A. suberitoides* (Broensted, 1934)	Massive; surface faintly hispid	Black outside, dark red inside	Very firm	Radiate, with loose spicule tracts	Styles 900–1100 × 15–23
*A. tentum* Kelly-Borges & Bergquist, 1994	Globular or sub-spherical; surface microscopically hispid	Different shades of brown outside, brown yellow inside	Firm	Large, loose tracts of megascleres in the choanosome, replaced in the outer region by intermediate spicules; superficial palisade of small tylo- and subtylostyles	Strongyloxeas I 980–2572 × 21–42; II 416–1298 × 10–21; Tylostyles 104–198 × 5–8; Styles or subtylostyles 187–441 × 8–13
*Aaptos lobata* sp. n.	Globular, sub-spherical	Yellow, dark orange, brown	Hard (preserved)	Radiate tracts of larger megascleres protrude towards the surface; intermediate and small spicules, abundant in the outer part, concur to the hispidation	Strongyloxeas: 810–993.91(±119.38)-1320 × 10–19.84(±3.84)-30; Intermediate megascleres: 405–540.91(±107.64)-750 × 7.5–11.53(±4.05)-25; Small megascleres 145–264.87(±65.20)-395 × 2.5–4.91(±1.43)-7.5

######### Etymology.

The name refers to the multi-lobate organisation of the sponge.

######### Remarks.

The genus *Aaptos* Gray, 1867, according to van Soest et al. (2016), encompasses in total 24 valid species, 10 of which distributed in the tropical Indo-Pacific and adjacent areas (Table 2). The descriptions are usually based on the very few diagnostic features detectable in the genus, making it difficult to differentiate species (Kelly-Borges and Bergquist 1994). The radial skeleton, the arrangement of the megascleres and the spicule morphology, being quite uniform within the genus, are seldom accurately described (Kelly-Borges and Bergquist 1994). Therefore, the importance of other morphological characters useful to differentiate species, such as colour, collagen distribution in the cortex, shape and arrangement of megasclere tracts, presence of interstitial spicules, is greatly emphasised (Kelly-Borges and Bergquist 1994). Recently, Carvalho et al. (2013) stressed the importance of other morphological aspects as main characters for the species distinction in the genus, such as external morphology, colour, shape and size of the megascleres, ectosomal spicules arrangement (palisade or bouquets).

The skeletal organisation of *Aaptos lobata* sp. n. is comparable with that of the type species of the genus, the Atlantic-Mediterranean *Aaptos aaptos* (Schmidt, 1864) (see van Soest 2002). *Aaptos lobata* sp. n. has been compared with all the congeneric species and especially with those recorded from the Indo-Pacific and adjacent areas, whose characteristics are reported in Table 2. *Aaptos ciliata* (Wilson, 1925) has spicules different in size and shape; in particular, the ectosomal styles are longer (1,100–1,300 × 4 µm). The species *A. conferta* Kelly-Borges & Bergquist, 1994, is an encrusting sponge, black outside and yellow inside, that has oxeas as additional spicules, whereas *A. globosa* Kelly-Borges & Bergquist, 1994 differs in colour (dark red outside and yellow inside) and in the skeletal organisation, since choanosomal tracts are thick and ramified under the surface and the intermediate megascleres form tracts. *Aaptos horrida* (Carter, 1886) and *A. nuda* (Kirkpatrck, 1903) have oxeas as megascleres instead of strongyloxeas; *A. laxosuberites* (Sollas, 1902) is encrusting, white in alcohol and has strongyloxeas and long tylostyles as megascleres. *Aaptos niger* Hoshino, 1981 is a black, massive sponge, usually embedding exogenous material; while *A. rosacea* Kelly-Borges & Bergquist, 1994, is red outside and yellow inside and differs from the new species in skeletal arrangement and size of spicules. The species *A. suberitoides* (Brøndsted, 1934), black outside and dark red inside, has a very simple skeleton of styles only, while *A. tenta* Kelly-Borges & Bergquist, 1994, brown in colour, has a peculiar skeletal arrangement and different spicules. Since no species in this vast geographic area matches with the characters of our specimens, we decided to erect a new species.

##### Order Tethyida Morrow & Cárdenas, 2015

###### Family Tethyidae Gray, 1848

####### Genus *Tethytimea* de Laubenfels, 1936

######## Tethytimea tylota

Taxon classificationAnimaliaTethyidaTethyidae

(Hentschel, 1912)

Figure 3

Donatia tylota Hentschel, 1912: 317.

######### Material examined.

BU-98, 23/03/2000, Lekuan II (Bunaken Island), 5 m depth. BU-289, 17/05/2001, Raymond’s Point (Bunaken Island), unknown depth. BU-533, 21/06/2004, Bualo (Manado Tua Island), about 8 m depth. BU-545, 23/06/2004, Raymond’s Point (Bunaken Island), about 20 m depth. BU-562, 26/06/2004, Bualo (Manado Tua Island), unknown depth.

######### Description.

**Figure 3.**
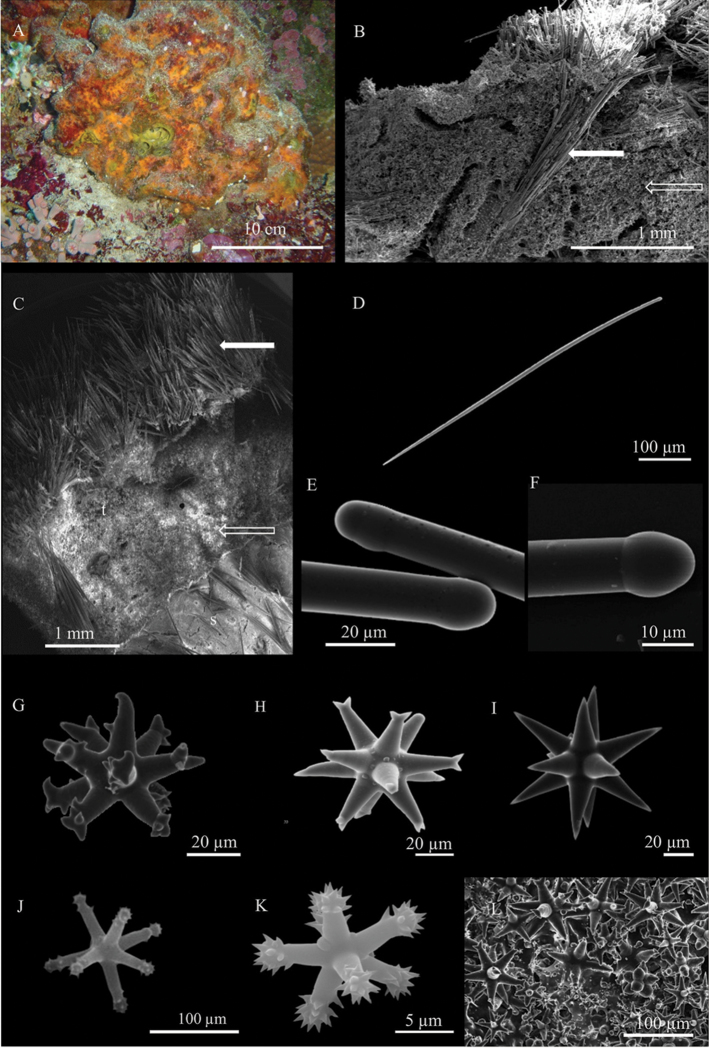
*Tethytimea tylota* (Hentschel, 1912) **A** specimen *in situ* (BU-562) **B** cross section showing bundles of big tylostyles (full arrow) and the microscleres (empty arrow) **C** SEM image showing fans of small tylostyles (full arrow) and microscleres (empty arrow) of *T. tylota* (**t**), below the sponge *Stelletta* sp. n. (**s**) involved in the association **D** small tylostyle **E, F** heads of tylostyles **G–I** oxyspherasters **J, K** tylasters **L** groups of microscleres.

Encrusting sponge 3–6 mm thick; the largest examined specimen (BU-289) is approximately 10 cm in diameter. The consistence is firm; the body of the sponge lacunose. The surface is irregular, with extended verrucous areas covered by sand and largely colonised by epibiotic ascidians, algae and hydroids (Fig. 3A). In the microscopic observation, the surface appears micro-hispid. The colour of living specimens is orange; when preserved, the sponge becomes yellowish-green.

**Skeleton.** *Tethytimea tylota* does not have a distinguishable ectosomal skeleton or a proper cortex; the choanosomal skeleton is formed by bundles of big tylostyles of 100–200 µm directed outwards (Fig. 3B). Close to the surface, these main bundles support fans of small tylostyles hispidating the sponge surface (Fig. 3B, C).

**Figure 4.**
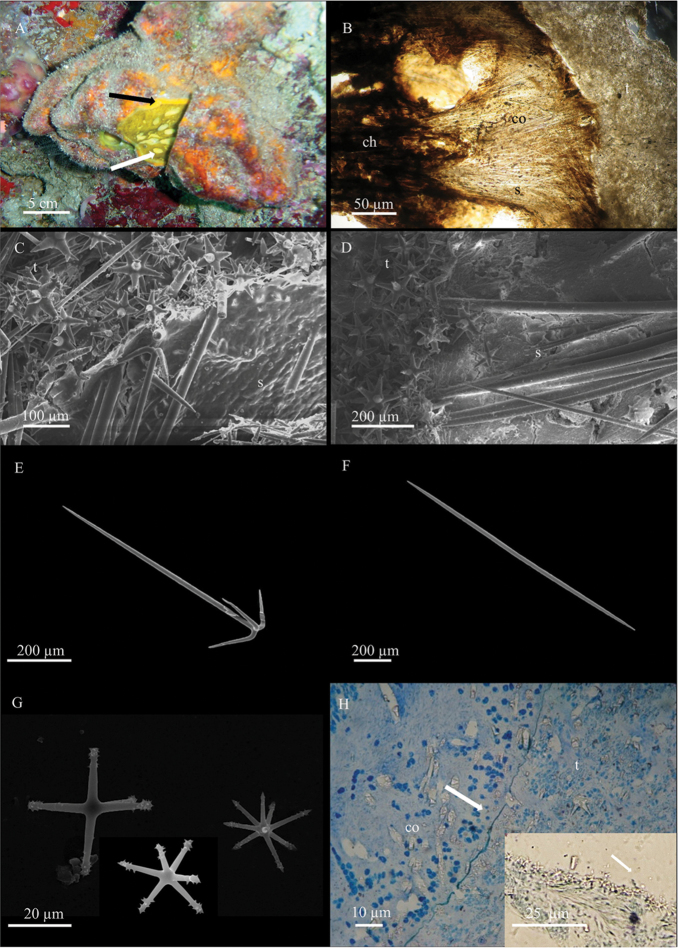
*Stelletta tethytimeata* sp. n. **A** specimen *in situ* (BU-533), partially cut to put in evidence the association with *Tethytimea tylota*. The black arrow indicates the thin layer of the external sponge (*T. tylota*, orange) while the white arrow indicates *S. tethytimeata* sp. n. **B** paraffin-embedded section of *T. tylota* (**t**) and *S. tethytimeata* sp. n. (**s**) **co** and **ch** indicate, respectively, the cortex and the choanosome of *S. tethytimeata* sp. n. **C** cross section showing triaenes close to the boundary between *T. tylota* (**t**) and *S. tethytimeata* sp. n. (**s**) **D** bundles of oxeas reaching the boundary between *T. tylota* (**t**) and *S. tethytimeata* sp. n. (**s**) **E** anatriaene **F** oxea **G** micrasters **H** histological preparation showing the cortex (**co**) of *S. tethytimeata* sp. n. The arrow points to the collagenous layer between *S. tethytimeata* sp. n. and *T. tylota* (**t**). The inset shows the layer of tylasters of *T. tylota* (arrow).

**Spicules.** Megascleres are straight tylostyles with a slightly developed head (Fig. 3D). They can be distinguished into two size classes (Fig. 3E, F); tylostyles I measure 930 - (1,104.8 ± 146.7) - 1,339 × 12.5 - (17.8 ± 3.4) - 25 µm; tylostyles II (Fig. 3D) measure 490 - (576.6 ± 72.5) - 660 × 5 - (6.6 ± 2.0) - 10 µm and form the superficial fans that protrude out of the surface; microscleres are two kinds of asters (Fig. 3G–K). Oxyspherasters (Fig. 3G–I) with thick ramified or rounded, often bifurcated rays, measuring 65 - (122.5 ± 39.6) - 200 µm. Tylasters with rays variable in length ending with apical groups of spines variable in number (Fig. 3J, K); they measure 7.5 - (11.1 ± 1.9) - 16.3 µm. Microscleres are abundant throughout the sponge, but more concentrated close to the surface (Fig. 3L), where the smallest tylasters form a thin, continuous layer (Fig. 4H, inlet).

######### Remarks.

This sponge was exclusively found as epizoic on *Stelletta tethytimeata* sp. n. (see below). It has been attributed to *T. tylota* for its skeletal organisation, made of bundles of main tylostyles supporting superficial fans of small tylostyles, the superficial layer of tylasters (present also in the holotype), the size and shape of megascleres and microscleres (Sarà 2002). The genus *Tethytimea* is monospecific and *T. tylota* was found at Aru Island (Indonesia). This is the first record of this species since the original description (Hentschel 1912). In the revision of the genus (based on the re-examination of the type material), Sarà (2002) confirmed the presence in the holotype of very rare spheres; these spicules were not detected in the present specimens as in the paratype (Sarà 2002).

It is interesting to note that the holotype of *T. tylota* was encrusting on a stone and in association with another sponge (Sarà 2002).

######### Remarks on the association.

See below.

##### Order Tetractinellida Marshall, 1876

###### Family Ancorinidae Schmidt, 1870

####### Genus *Stelletta* Schmidt, 1862

######## Stelletta tethytimeata

Taxon classificationAnimaliaTetractinellidaAncorinidae

Calcinai, Bastari, Bertolino & Pansini sp. n.

http://zoobank.org/8C01D0F2-326D-4C50-827F-706CF3D6EAF6

Figure 4

######### Material examined.

Holotype: MSNG 60136, BU-289, 17/05/2001, Raymond’s Point (Bunaken Island), unknown depth. Paratype: MSNG 60137, BU-562, 26/06/2004, Bualo (Manado Tua Island), unknown depth.

######### Other material.

BU-533, 21/06/2004, Bualo (Manado Tua Island), about 8 m depth. BU-545, 23/06/2004, Raymond’s Point (Bunaken Island), about 20 m depth. BU-98, 23/03/2000, Lekuan II (Bunaken Island), 5 m depth.

######### Diagnosis.

Massively rounded yellow sponge; the colour changes after fixation. Megascleres are anatriaenes with characteristic bending and a single type of oxeas; microscleres are represented by a heterogeneous set of tylasters and oxyasters.

######### Description.

The sponge is light yellow-lemon *in vivo* (Fig. 4A); the colour changes in the preserved specimens, becoming dark-brown to blackish. It is almost totally covered by the associated epibiotic species *T. tylota* (see above), with the exception of the oscula that, protruding from the surface of *T. tylota*, are clearly distinguishable for their different colour (Figs 3A, 4A). Since the external sponge *T. tylota* is thinly encrusting, most of the mass of the associated sponges is due to *S. tethytimeata* sp. n. that can be as large as 10 cm across (Fig. 4A, B).

**Skeleton**. The cortex is a collagenous layer 400-700 µm thick (Fig. 4B); the triaenes have their clades tangential to the surface and sometimes protrude from it (Fig. 4C), merging in the tissue of the epibiotic *T. tylota*. The choanosomal skeleton is formed by tracts of oxeas without a clear radial arrangement with microscleres scattered in between (Fig. 4D). Towards the sponge surface, the spicule density lowers and oxeas are more or less parallelly arranged (Figs 3C, 4B, D).

**Spicules.** Megascleres are anatriaenes (Fig. 4E), with straight, sharp-pointed rhabdome of 570 - (708.2 ± 119.3) - 800 × 10 - (15.7 ± 3.8) - 22.5 µm and clads of 80 - (113.4 ± 43.3) - 225 × 7.5 - (9.0 ± 2.6) - 12.5 µm with sharp tips and characteristic bending. Oxeas straight, fusiform, with sharp tips (Fig. 4F), sometimes modified into styles; they measure 1274 - (1514.5 ± 145.3) - 1950 × 20 - (24.5 ± 3.9) - 30 µm. Microscleres encompass a heterogeneous set of tylasters and oxyasters (Fig. 4G), with 4–9 rays, with spines along the rays or grouped at the extremities 20 - (27.2 ± 4.4) - 35 µm.

######### Etymology.

The name refers to the association with *Tethytimea tylota*.

######### Remarks.

*Stelletta tethytimeata* sp. n. is characterised by one type of triaenes and by a single category of oxeas. Out of the 146 species of *Stelletta*, distributed in all the oceans (van Soest et al. 2016), 49 are from the tropical Indo-Pacific area (van Soest 1994). However, they all differ from the new species in colour, skeletal organisation and especially in the spicule features. They show different categories of megascleres (oxeas of different sizes, plagio-, orto- and dico-triaenes) and microscleres. In particular, 10 species of the tropical Indo-Pacific *Stelletta* species present a single type of triaenes: *S. bocki* Rao, 1941, *S. brevioxea* (Pulitzer-Finali, 1993) and *S. cavernosa* (Dendy, 1916) have ortotriaenes; *S. brevis* Hentschel, 1909, *S. centroradiata* Lévi and Lévi, 1983, *S. centrotyla* Lendelfeld, 1907 and *S. herdmani* Dendy, 1905 have plagiotriaenes; S. herdmani var. robusta Thomas, 1979 has protriaenes, whereas *S. hyperoxea* Lévi and Lévi, 1983, *S. vaceleti* (Lévi and Lévi, 1983), *S. phialimorpha* Lévi, 1993 and *S. digitata* (Pulitzer-Finali, 1993) have dicotriaenes. Actually, *Stelletta tethytimeata* sp. n. is the only species of the genus in this area possessing anatriaenes (peculiar for the characteristic clad bending) and a single category of oxeas. It is therefore justified, based on the five specimens in association with *Tethytimea tylota* encountered in this region, to erect a new species.

######### Remarks on the association.

The associated specimens of *T. tylota* and *S. tethytimeata* are flat or cushion-shaped with big, rounded lobes and wide oscular structures (Figs 3A, 4A).

By superficial analysis, the two associated species could appear as a single large individual sponge. The external species (*T. tylota*) can be detached with difficulty from the internal one (*S. tethytimeata* sp. n.); the contact area may be observed in SEM images (Fig. 3C) and by histological preparations where the presence of a thin collagen layer of separation between the two species is detectable (Fig. 4B, H). Histological preparations clearly show the presence of the cortex of *S. tethytimeata* sp. n. made by a collagen layer up to 700 µm thick (Fig. 4B, H). In the cortex, collencytes are clearly visible and pigmentary cells are numerous (Fig. 4H).

The two associated species are quite common in North Sulawesi, always in association, generally in dim-light conditions, at a maximum depth of 20 m.

####### Genus *Rhabdastrella* Thiele, 1903

######## Rhabdastrella distincta

Taxon classificationAnimaliaTetractinellidaAncorinidae

(Thiele, 1900)

Figure 5

Coppatias distinctus Thiele, 1900: 56.

######### Material examined.

BU-560, 26/06/2004, Bualo (Bunaken Island), unknown depth. BU-575, 27/06/2004, Alung Bauna (Bunaken Island), 27 m depth.

######### Description.

**Figure 5.**
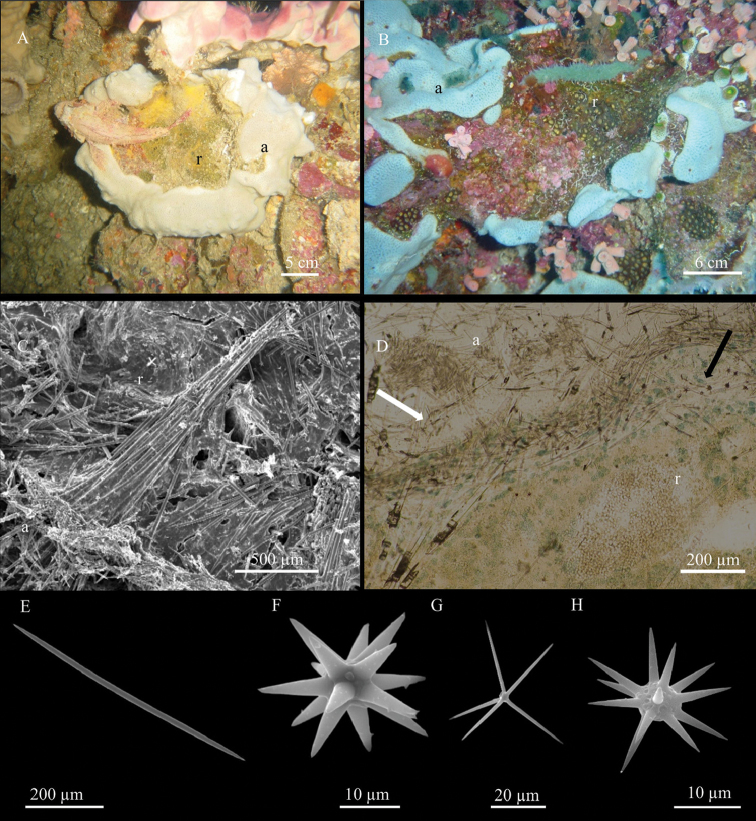
*Rhabdastrella distincta* (Thiele, 1903) **A, B** specimens *in situ* (**r**), partially covered by the epibiotic sponge Amphimedon cf. sulcata Fromont, 1993 (**a**) specimen of Figure 5A is BU-575, that of Figure 5B is BU-560 **C** SEM image of a cross section of *R. distincta* (**r**) showing the radial tracts of oxeas in proximity of the external part, **a** indicates the epibiotic sponge A. cf. sulcata **D** histological preparation of *R. distincta* (**r**) and A. cf. sulcata (**a**) showing spherasters (black arrow) in the peripheral part, and oxeas of *R. distincta* (white arrow) penetrating the tissues of A. cf. sulcata **E** oxea **F** spheraster **G** oxyaster **H** oxyspheraster.

The sponge has a massive and irregular shape, a large size, up to 50 cm in diameter, and was exclusively found partially covered by Amphimedon cf. sulcata (see below). In the part not covered by the epibiotic sponge, *R. distincta* is yellow-lemon (Fig. 5A), or dark green (Fig. 5B), turning black when cut or preserved. Wide oscular areas are often evident (Fig. 5A, B).

**Skeleton.** Spherasters are located in the outer part of the sponge, but do not form a real cortex (Fig. 5C, D). The choanosomal skeleton consists of scattered oxeas which tend to form radial tracts towards the peripheral part (Fig. 5C). Oxyasters and oxyspheraster are dispersed in the choanosome.

**Spicules.** Megascleres are fusiform oxeas (Fig. 5E) with rather sharp tips, 720 - (832.5 ± 65.7) - 990 × 10 - (13.3 ± 2.9) - 20 µm. Microscleres are spherasters of variable size, 12.5 - (29.5 ± 6.4) - 35 µm in diameter (Fig. 5F), with a large centre and thick rays with sharp or bifurcated tips; oxyasters (Fig. 5G) with small centre and thin rays, 35 - (49 ± 8.1) - 65 µm in diameter; oxyspherasters with well-developed centre (Fig. 5H), 10 - (15.1 ± 2.6) - 20 µm.

######### Remarks.

The Indonesian specimens fit with the description of *R. distincta* in having the same skeletal organisation (characterised by oxeas scattered in the inner part of the sponge and radially arranged close to the surface), absence of triaenes, spherasters in the peripheral part, oxyasters and oxyspheraster scattered in the choanosome. Spicule sizes are comparable to those of the type species that are fusiform oxeas of 850 × 25 µm, spherasters up to 40 µm, oxyasters up to 80 µm and oxyspherasters of 15 µm (see Uriz 2002). The principal difference with Thiele’s original description is that smooth microscleres were not observed and a real cortex is not detectable in the studied specimens.

This is the first record of the species since the original description of Thiele (1900) based on two specimens from Ternate, Indonesia.

######### Remarks on the association.

See below.

##### Order Haplosclerida Topsent, 1928

###### Family Niphatidae

####### Genus *Amphimedon* Duchassing & Michelotti, 1864

######## Amphimedon cf. sulcata

Taxon classificationAnimaliaHaploscleridaNiphatidae

Fromont, 1993

Figure 6

######### Material examined.

BU-560, 26/06/2004, Bualo (Bunaken Island), unknown depth. BU-575, 27/06/2004, Alung Bauna (Bunaken Island), 27 m depth.

######### Description.

**Figure 6.**
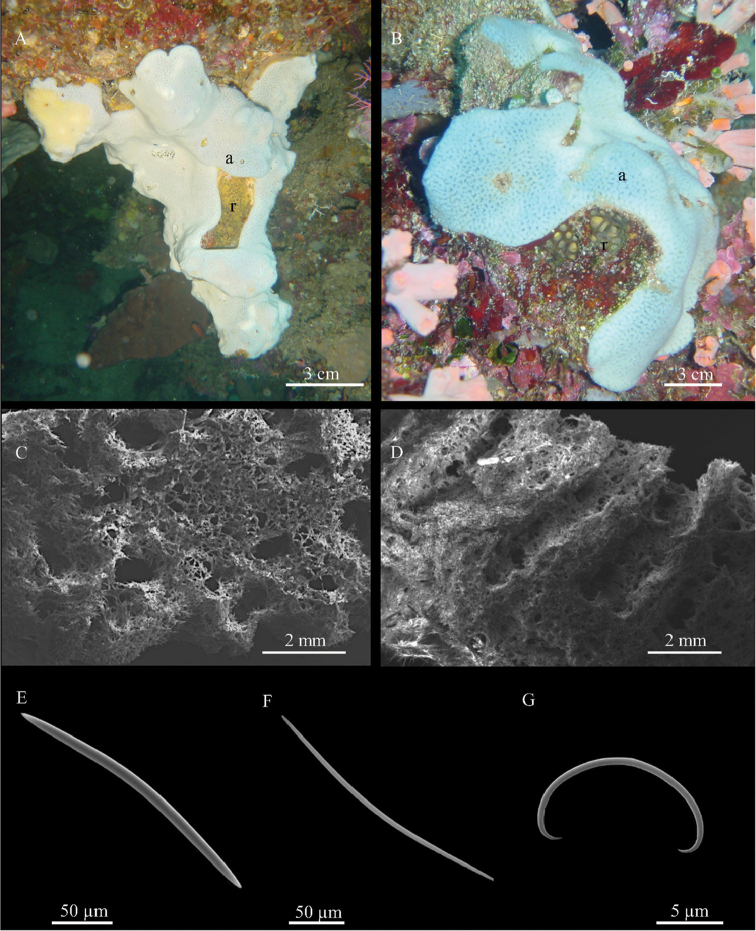
Amphimedon cf. sulcata Fromont, 1993 **A, B** specimens *in situ* (**a**), partially covering the associated sponge *Rhabdstrella distincta* (**r**), specimen BU-560a1 in Figure 6A, BU-560 in Figure 6B **C** SEM image of the ectosome **D** SEM image of the choanosome **E** oxea **F** thin oxea **G** sigma.

The sponge is flat, with a roundish contour, about 1 cm thick, without visible oscules. It is completely free of epibiotic organisms. Colour *in situ* may be greyish-white (Figs 5A, 6A) or pale cerulean (Figs 5B, 6B), off-white to greyish in the preserved state. The sponge shows ridges and grooves, covered by a very thin membrane, that give a typical convoluted or brain-like aspect to its surface (Fig. 6B).

**Skeleton**. The ectosomal skeleton is a reticulation of pauci-spicular tracts (3-4 spicules) (Fig. 6C) organised in quite regular triangular meshes with scarce spongin at the nodes. The choanosomal skeleton (Fig. 6D) is formed by a reticulation of multi-spicular tracts and round meshes of approximately 60 µm in diameter, with abundant scattered spicules. The spicule tract extremities barely protrude from the sponge surface, causing micro-hispidation.

**Spicules.** Megascleres are straight or slightly curved oxeas with sharp tips; they measure 125 - (188.9 ± 33.5) - 247.5 × 2 - (5.2 ± 3.4) - 12.5 µm (Fig. 6E); numerous thin oxeas are present (Fig. 6F); microscleres are very thin, C-shaped, sigmas 10 - (12.9 ± 1.5) - 15 × ≤ 1µm (Fig. 6G).

######### Remarks.

The sponge here described has a skeleton organisation fitting with the diagnosis of the genus *Amphimedon* that is characterised by an ectosomal skeleton of tangential fibres forming meshes, covered by a thin membrane and by a choanosomal skeleton formed by a plumose, irregular reticulation of multispicular tracts (Desqueyroux-Fáundez and Valentine 2002).

Our specimens are similar to *A. sulcata*, especially for the very characteristic surface: “meandering parallel ridges, interspersed with spaces, give a convolute or brain-like appearance to the surface” (Fromont 1993), for the thin membrane covering the ridges and the absence of abundant spongin.

Among the Indo-Pacific species of *Amphimedon*, only *A. sulcata* has sigmas similar in size (13 - (15.9) - 16.9 µm) to our specimens, but its oxeas (122 - (139) - 153 × 3 - (4.5) - 5.3 µm) are smaller than those we observed. Another difference is in the colour: “mauve alive, cream or fawn in alcohol” in *A. sulcata* (Fromont, 1993).

######### Remarks on the association.

Amphimedon cf. sulcata is not tightly attached to *Rhabdastrella distincta*, and the two sponges can be separated rather easily. Frequently, wide areas of *R. distincta* are not covered by the outer sponge (Figs 5A, B, 6A, B), and exhalant and probably also inhalant parts of *R. distincta* are in these portions, free from the epibiont.

In the boundary between the two sponges, a thin collagenous layer is present. Both in the histological preparations and in SEM images, the oxeas of *R. distincta* are clearly visible, protruding out of the surface and penetrating inside the tissues of the external sponge (Fig. 5C, D), as it is usual in similar associations (Ávila et al. 2007). This association was frequently observed in North Sulawesi, usually below a depth of 30 m.

######## Amphimedon anastomosa

Taxon classificationAnimaliaHaploscleridaNiphatidae

Calcinai, Bastari, Bertolino & Pansini sp. n.

http://zoobank.org/768365CA-8FBA-4660-A3B9-90E76B42A940

Figure 7

######### Material examined.

Holotype: MSNG 60138, PH-58, 17/01/2005, Tiwoho (Bunaken Island), about 20 m depth.

######### Diagnosis.

Dark green, highly branched sponge with an irregular ectosomal skeleton of rectangular, paucispicular meshes and multispicular choanosomal fibres, forming an irregular reticulation. Oxeas are mucronate.

######### Description.

**Figure 7.**
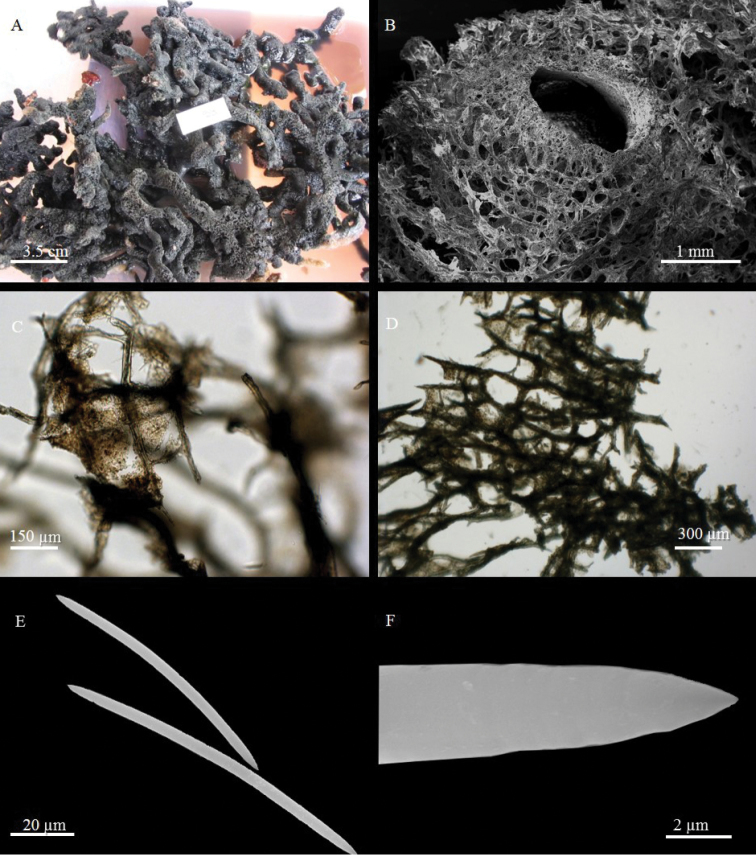
*Amphimedon anastomosa* sp. n. **A** The holotype just after collection **B** Sponge surface with the round opening of a symbiotic barnacle **C** ectosomal skeleton **D** choanosomal skeleton **E** oxeas **F** magnification of an oxea tip.

Highly branched sponge (Fig. 7A) with repent habit. Anastomosing branches are flattened, 4–8 mm in diameter, creeping over the substrate. Colour *in situ* is dark green to dark brown, greenish in alcohol or in the dried state. Consistence soft and brittle; the sponge easily crumbles when dried. Surface slightly rough, irregular; when the transparent membrane is preserved, it gives a smooth appearance at the macroscopic observation. Oscula not visible. Numerous barnacles are embedded in the sponge tissue, with only their openings free (Fig. 7B).

**Skeleton.** The ectosomal skeleton is an irregular reticulation of rectangular meshes 120–150 µm, up to 190–250 µm in diameter, formed by fibres 20–40 µm thick (Fig. 7B, C). Fibres are cored by 4–6 spicules. In the well-preserved parts of the sponge, a thin dermal membrane covers the surface. When the membrane is damaged, the sponge surface is microhispid due to protruding fibres. The choanosomal skeleton (Fig. 7D) is irregular, formed by primary multispicular (approximately 10 spicules) fibres, about 60 µm thick, directed towards the surface; secondary fibres are 20–35 µm in diameter. Secondary and primary fibres create an irregular reticulation of more or less circular meshes 170–300 µm across. Spongin is not abundant.

**Spicules.** Megascleres are oxeas slightly curved, with sharp tips (Fig. 7E, F), 97 - (111.6 ± 6.7) - 122.4 × 2.6 - (4.5 ± 1.2) - 5.2 µm.

######### Etymology.

The name refers to the habitus of the sponge, characterised by anastomosing branches.

######### Remarks.

The species described here may be attributed to the genus *Amphimedon* due to its skeleton characteristics. Out of the 54 species of *Amphimedon* hitherto described (van Soest et al. 2016), only two (*A. denhartogi* de Voodg, 2003 and *A. elastica* (Kieschnick, 1898) are present in Indonesia, whereas 30 have been recorded in the Indo-Pacific region. *Amphimedon denhartogi* and *A. elastica* differ from *A. anastomosa* sp. n. in their skeletal organisation and general morphological characters. The species *A. denhartogi* is green in life, like *A. anastomosa* sp. n., but it has an erect, flabellate shape and star-shaped oscula; moreover, it has strongyles as spicules. In contrast, *A. elastica* is a single-tube yellow-brownish sponge with a wide apical osculum (11 mm in diameter) and smooth surface; spicules are oxeas of 90–100 µm. Also, the other Indo-Pacific species show significant differences with *A. anastomosa* sp. n.; *A. aculeata* Pulitzer-Finali, 1982 is a vase-shaped sponge with conical projections on the surface and strongyles as spicules, whereas *A. aitsuensis* (Hoshino, 1981), described from Japan, is a massive sponge, grey in colour and with oxeas of two distinct size categories (thick oxeas of 132–148 × 7–9 µm and thin oxeas of 115–135 × 4–6 µm). *Amphimedon alata* Pulitzer-Finali, 1996 has oxeas of 100–130 × 7–11.5 µm and peculiar, small, wing-shaped toxas (11–50 µm); *A. brevispiculifera* (Dendy, 1905) is an erect sponge light-brown in the dry state; it is digitate or flabellate, with evident large oscula; it differs from *A. anastomosa* sp. n. also for its stout primary fibres 164 µm thick. The two species *A. chinensis* and *A. flexa* have been described by Pulitzer-Finali (1982) from Hong Kong; *A. chinensis* differs from the new species for the orange colour, the presence of oscula arranged in a single row and the larger oxeas (125–145 × 8–9.5 µm), while *A. flexa* is plurilobate with oscula on top of the lobes; its primary fibres, slightly thicker than those of the new species, create larger meshes from 300 to 900 µm across. The species *A. chloros* Ilan et al., 2004 is green, like *A. anastomosa* sp. n., but cushion-shaped, with oxeas that usually become strongyloxeas. In contrast, *A. conferta* Pulitzer-Finali, 1996 is sub-cylindrical, brown in life, cream in the dry state, with ectosomal tracts 75 µm in diameter; spicules are oxeas longer and thicker (140–160 × 7–9 µm) than those of *A. anastomosa* sp. n., with frequent stylote modifications. *Amphimedon cristata* Pulitzer-Finali, 1996 is sub-cylindrical, violet in colour and rigid, with an apical osculum; it has large oxeas (230–370 × 11–18 µm) with blunt extremities. Other three species of *Amphimedon* have been described by Helmy and van Soest (2005) from the Red Sea: *A. dinae*, *A. jalae*, *A. hamadai*. *Amphimedon dinae* is a brown, massive sponge with oscula 2-4 mm wide and very thin and short oxeas (52–61 × 1–1.5 μm); *A. jalae* is massive, cushion-shaped, with large oxeas (100–170 × 4–6 μm) and choanosomal rounded meshes of 600–800 μm. *Amphimedon hamadai* is brown, irregularly lobated, with very short oxeas (48–60 × 2–3 μm), while *A. delicatula* (Dendy, 1889) is erect, bushy, yellow in colour and with stout fibres 126 µm thick and very slender, slightly curved oxeas (98 by 3.5 µm). *Amphimedon lamellata* Fromont, 1993 is a lamellate, erect sponge, pale pink in colour; with a reticular choanosomal skeleton and two types of oxeas differing in thickness (111–130 × 2.5–4.4 µm and 105–126 × 1.3–2.3 µm); *A. massalis* (Carter, 1886) is massive, yellow in the basal portion, dark brown-red on the surface, with vents “on monticular elevations” and oxeas measuring 155 × 6 µm. *Amphimedon navalis*, *A. rubida*, *A. rubiginosa* and *A. spinosa* have been described by Pulitzer-Finali (1993) from Kenya. *Amphimedon navalis* is a cushion-shaped sponge, dark blue and violet in colour, with blunt oxeas (160–210 × 11–15 µm); *A. rubida* is cylindrical, red brownish, with meshes of 220–360 µm across and oxeas measuring 185–230 × 11.5–18 µm. *Amphimedon rubiginosa* has a massive shape with elevated oscula and a skeletal organisation with ill-defined plurispicular tracts. *Amphimedon spinosa* has a tubular shape and fibres cored by single spicules, while *A. paraviridis* Fromont, 1993 is encrusting or ramose, green-olive in life, with primary fibres of 50–160 µm and secondary of 20–50 µm, thicker than those of the new species. Moreover, abundant oxeas (133–151 × 3.9–8.0 µm) are scattered in between the fibre reticulation (absent in *A. anastomosa* sp. n.). *Amphimedon queenslandica* Hooper & van Soest, 2006 is a blue-grey and green sponge with an encrusting base from which lobate or digitate portions rise. Unlike the new species, it has unispicular fibres. *A. robusta* (Carter, 1885) is a branching-digitate, orange sponge with oscula located on one side; *A. rudis* Pulitzer-Finali, 1996 is violet-brownish, with blunt and very stout oxeas (360–420 × 10–12.5 µm). *Amphimedon strongylata* Pulitzer-Finali, 1996 is sub-cylindrical, grey in colour, with strongyloxeas as megascleres; *A. subcylindrica* (Dendy, 1905) is a cylindrical sponge with reptant habit; it has a smooth surface and oscula with prominent rims; its fibres are cored by a high number of spicules (slightly longer (140 × 8 µm) oxeas), without visible spongin. *Amphimedon sulcata* Fromont, 1993 is a small, globular sponge with oxeas of 122–153 × 3.0–5.3 µm and C-shaped sigmas as microscleres. Finally, *A. zamboangae* (Lévi, 1961), which is green in colour, has a velvety surface, thick fibres (130 µm) and two types of oxeas (120–150 × 4–6 µm and 120–130 × 3 µm).

“*Amphimedon* differ from other Niphatidae in having an optically smooth, but microscopically microtuberculate fibrous superficial skeleton, usually with abundant spongin, and lacking microscleres” (Hooper and van Soest 2006). Because of the slight differences between *Amphimedon* and *Niphates* (Desqueyroux-Fáundez & Valentine 2002), all the Indo-Pacific species of the latter genus were also checked. All these species of *Niphates* differ from the new species in shape, colour and skeletal organisation. The most similar species, in terms of the branched shape, is *N. aga* (de Laubenfelds, 1954), but it has a confused ectosomal skeleton and longer oxeas (175–180 µm). *Amphimedon anastomosa* sp. n. is well characterised by its growth form and colour. Since no species in this vast geographic area matches with our specimen, we are justified to erect a new species.

####### Genus *Niphates* Duchassaing & Michelotti, 1864

######## Niphates laminaris

Taxon classificationAnimaliaHaploscleridaNiphatidae

Calcinai, Bastari, Bertolino & Pansini sp. n.

http://zoobank.org/4E0827B5-02C7-45D4-8456-78E0F8AE1B31

Figure 8

######### Material examined.

Holotype: MSNG 60139, PH-47, 17/01/2005, Tiwoho (Bunaken Island), 20 m depth.

######### Diagnosis.

Lamellate, azure-violet sponge, with differentiated inhalant and oscular faces. Skeleton is a regular reticulum of primary and secondary fibres, with superficial brushes hispidating the surface; megascleres are straight and sinuous oxeas. Microscleres are sigmas.

######### Description.

**Figure 8.**
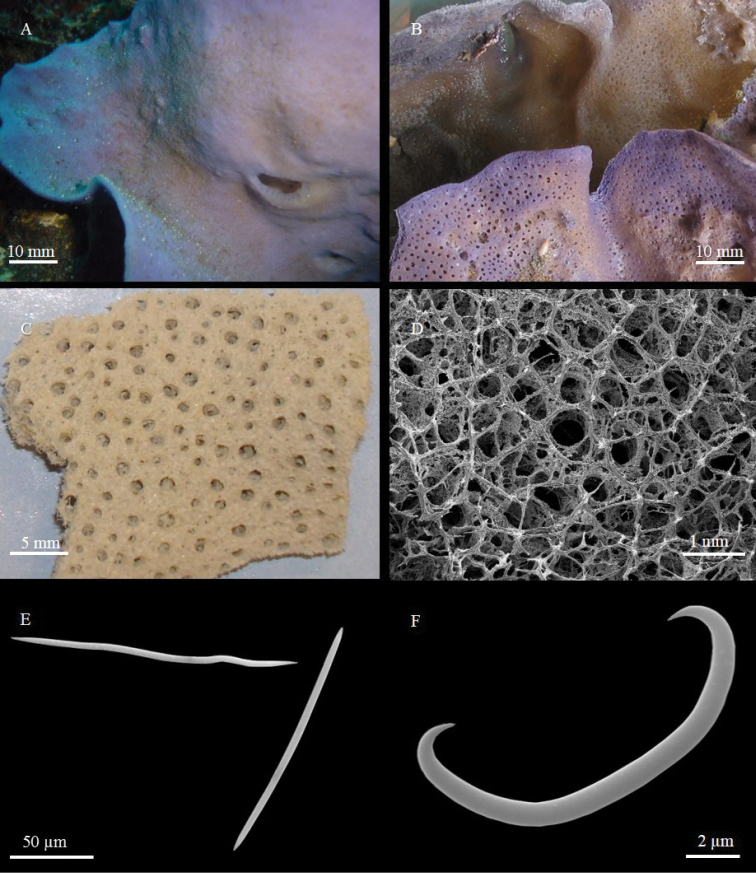
*Niphates laminaris* sp. n. **A** holotype *in situ* **B, C** holotype freshly collected showing the exhalant side of the sponge **D** sponge skeleton on the exhalant side with in evidence the choanosomal ascending tracts protruding through the surface and the vents **E** sinuous and straight oxeas **F** sigma.

The sponge is a thin, irregular, folded lamina, attached to the substrate in few points (Fig. 8A); its rim is more or less rounded, not regular (Fig. 8B). The holotype consists in alcohol-preserved fragments, collected from a bigger specimen (Fig. 8A, B). The largest observed specimen is approximately 8 × 4 cm long and 2 mm thick. The colour in life is azure-violet in the part exposed to light and beige on the shadowed side (Fig. 8B). The sponge becomes white-bluish when dried. Consistence soft, slightly elastic. The aspect of the two sides of the laminar sponge is different: roundish vents, 700–1,300 µm in diameter, most probably acting as oscula, are concentrated on the excurrent side (Fig. 8C); on the opposite side, a thin dermal membrane, pierced by numerous pores, covers several smaller apertures, not visible to the naked eye (Fig. 8D). In the dried state, spicule brushes and small ridges (made by tracts of tangential oxeas connecting the brushes) create a microconulose surface, visible also to the naked eye, in both sides of the sponge.

**Skeleton.** The ectosomal skeleton is a reticulation of multispicular tracts (30–60 µm thick) forming polygonal (mostly quadrangular) meshes 340–900 µm in diameter, with brushes of spicules at the nodes (Fig. 8D). The choanosomal skeleton is a not very regular reticulation, with elongated, almost rectangular meshes 400–800 µm across and empty spaces. The spicule tracts may be divided into ascending primary tracts, 55–100 µm thick, and secondary tracts, 25–35 µm thick, with a more or less transverse arrangement. The extremities of the ascending tracts protrude through the surface, forming brushes (Fig. 8D). Very numerous pigmented (green) cells and abundant spicules, both megascleres and microscleres, are dispersed in the ectosome and choanosome.

**Spicules.** Oxeas slightly curved or sinuous, rarely straight, with acerate tips (Fig. 8E). They measure 150.8 - (163.37 ± 7.0) - 176.8 × 2.5 - (3.7 ± 1.1) - 5.2 µm. Sigmas C-shaped, sometimes with a part of the shaft almost straight (Fig. 8F). They measure 13 - (17.0 ± 3.18) - 23.4 µm × 1 µm.

######### Etymology.

The name refers to the lamellate shape of the sponge.

######### Remarks.

The new species clearly belongs to the family Niphatidae for the presence of multispicular fibres in the ectosome and to the genus *Niphates* for the skeletal organisation. The genus *Niphates* includes sponges with “Surface conulose to spiny [….] produced by primary longitudinal fibres ending on surface” (Desqueyroux-Faúndez and Valentine 2002). The ectosomal skeleton is a tangential network of secondary fibres, obscured by protruding tufts of primary fibres. Microscleres are rare sigmas (Desqueyroux-Faúndez and Valentine 2002). However, other species of the genus (e.g. *Niphates nitida* Fromont, 1993) have a smooth surface as the new species.

*Niphates laminaris* sp. n. is characterised by a non-spiny, rather irregular, microconulose surface and by a choanosomal skeleton with a reticulation of primary and secondary tracts. Microscleres are numerous. In the Indo-Pacific area, only *N. nitida* has sigmas. However, *N. nitida* is a sponge with repent habit, with oscula located at the top of small erect lobes; a choanosomal fibrous reticulation with round or triangular meshes (104–146 µm) and oxeas measuring 128 × 5.6 µm. Therefore, it substantially differs from *Niphates* sp. n; all other *Niphates* in the area differ from the new species for the absence of sigmas and for other significant features listed below. *Niphates olemda* (de Laubenfelds, 1954) is a blue, or pink tubular sponge with small oxeas (92–100 × 2–3 µm), while *N. aga* (de Laubenfelds, 1954) is ramose with superficial projections, a confused ectosomal skeleton and straight and large oxeas (175–180 × 5 µm). *Niphates cavernosa* Kelly-Borges & Bergquist, 1988 is a massive, creeping and branching sponge, violet in life, with two categories of oxeas differing in thickness (oxeas I: 5–10 µm thick; oxeas II: 2–4 µm); *N. furcata* (Keller, 1889) is green, erect, branching, with rather short oxeas (100 × 12 µm). *Niphates hispida* Desqueyroux-Fáundez, 1984 is a hard and incompressible sponge with very small oxeas (60-80 × 2-4 µm), consisting of a series of coalescent, cylindrical tubes arising from a massive common base. *Niphates mirabilis* (Bowerbank, 1873) is an ochre-pinkish sponge with a unispicular ectosomal reticulation, while *N. obtusispiculifera* (Dendy, 1905) is a branching, cylindrical sponge with strongyles as megascleres. *Niphates plumosa* (Bowerbank, 1876) is fawn-coloured and has a peculiar, stipitate and fan-shaped growth form with only oxeas as spicules. *Niphates rowi* Ilan et al., 2004 is the species most similar to the new species. Its ectosomal skeleton is a reticulation of fibres creating quadrangular meshes which are smaller than those of *Niphates laminaris* sp. n. (70–115 µm). In addition, the choanosomal reticulation of *N. rowi* has rectangular meshes which are smaller (115–200 µm) than those of *Niphates laminaris* sp. n., whereas the oxea size is similar (115 - (140) - 170 × 5.5 - (6.5) - 7.5 µm). In conclusion *N. rowi*, which is an encrusting sponge, differs from *Nipahtes laminaris* sp. n. in the growth form, the absence of sigmas and sinuous oxeas and in the size of the ectosomal and choanosomal meshes.

#### Subclass Keratosa

##### Order Dictyoceratida

###### Family Irciniidae Gray, 1867

####### Genus *Psammocinia* Lendenfeld, 1889

######## Psammocinia alba

Taxon classificationAnimaliaDictyoceratidaIrciniidae

Calcinai, Bastari, Bertolino & Pansini sp. n.

http://zoobank.org/2304C2B3-8156-4163-AC33-0AEC55EBADEE

Figure 9

######### Material examined.

Holotype: MSNG 60140, PH-41, 14/01/2005, Timur (Bunaken Island), 22 m depth.

######### Diagnosis.

Lobate, white sponge with oscular cavities at the top of the lobes. Thin armoured surface with sand and foreign spicules. Slightly fasciculated fibres, not very dense.

######### Description.

**Figure 9.**
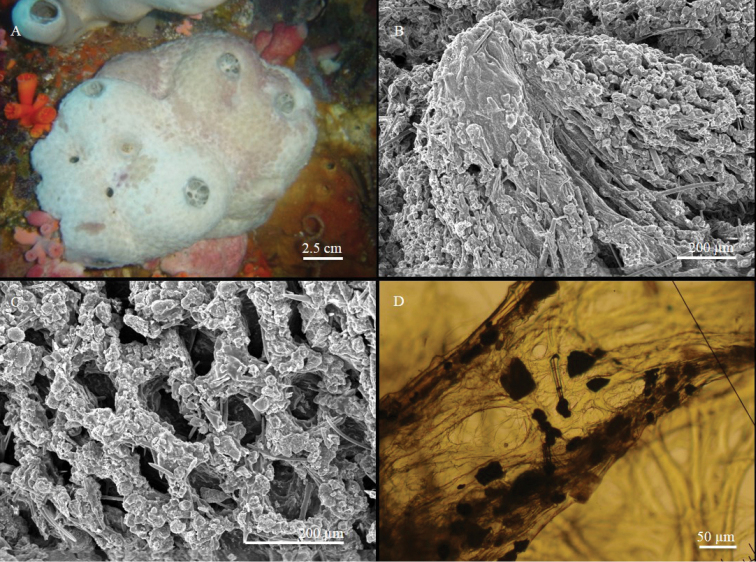
*Psammocinia alba* sp. n. **A** the sponge *in situ* **B** a small conule at SEM **C** reticulation made of sand grains and foreign spicules **D** primary fibres cored with foreign material and, on the right, secondary fibres free from inclusions.

Massive, lobate sponge with flush, roundish oscular cavities (about 1.5 cm) where the excurrent canals converge, located at the top of the lobes (Fig. 9A). The deposited holotype consists of fragments 3 × 1.5 cm, coming from a larger specimen approximately 15 cm across (Fig. 9A).

The colour in life is white outside (Fig. 9A) and cerulean inside; it becomes light cerulean after collection and beige after preservation in alcohol. Surface characterised by numerous small conules, 0.5–1 mm high and 2 mm apart, united by ridges (Fig. 9A, B). Consistence soft, but elastic, difficult to tear apart.

**Skeleton.** The surface is covered by a thin reticulation of sand and foreign spicules, forming regular, more or less circular, meshes 100 µm in diameter (Fig. 9C), well visible in the stereo-microscope. The density of the fibres is moderate. The primary fibres of the choanosome are slightly fasciculated (Fig. 9D), about 80 µm thick and cored with foreign debris and a few foreign spicules. The secondary fibres are thinner (20 µm in diameter) and free from inclusions (Fig. 9D). The size of the ovoid meshes ranges from 50 × 80 to 57.5 × 115 µm; a few smaller meshes, 30 × 55 µm, are also present. Filaments, 2.5 µm thick, are numerous and dense.

######### Etymology.

Referring to the white colour in life.

######### Remarks.

Our species is attributed to *Psammocinia* due to the presence of a surface armoured by sand and foreign spicules and to the reticular skeleton of primary and secondary fibres.

According to van Soest et al. (2016), 25 species of *Psammocinia* are known in total. Most of them have been described from New Zealand and South Korea and only one from Brazil.

*Psammocinia bulbosa* Bergquist, 1995 from New Caledonia and *P. lobatus* Sim & Lim, 2002 from Korea are the most similar species to *Psammocinia alba* sp. n. *Psammocinia bulbosa* is a massive, repent sponge with quite long oscular fistules. Its surface is covered by small conules 0.5–1 mm high and has a sandy crust up to 1 mm thick. The skeleton is formed by primary fibres giving rise to columns up to 700 µm long and secondary fibres 30–50 µm in diameter. The main differences to our species are the presence of fistules, a distinctive characteristic of *P. bulbosa*, and thicker fibres. *Psammocinia lobatus*, lobate in shape, has a surface covered by conules 1–2 mm high and 2–5 mm apart. Both primary and secondary fibres (60–10 µm thick) are comparable in size with our species. The main differences to *P. alba* sp. n. are the colour (dark brown, black), the presence of sharp conules and the small amount of foreign material present in the fibres. From New Zealand, the following species have been described: *P. beresfordae* Cook & Bergquist, 1996, formed by a compact base with broad-based fistules with an apical osculum 3–7 mm in diameter and primary fibres 120 µm thick; *P. verrucosa* Cook & Bergquist, 1996, a small, massive sponge with a very characteristic surface with rounded lamellae supported by skeletal fibres and a reticulate pattern; *P. hirsuta* Cook & Bergquist, 1998, formed by a coalescent group of digitate structures or lobes, with long, cylindrical fistules and a thick (400 µm) superficial sand layer; *P. charadrodes* Cook & Bergquist, 1998, a massive sponge with very long, rounded conules and very thick (till 1086 µm) primary fibres; *P. papillata* Cook & Bergquist, 1998, a massive, compact sponge with a coarsely conulose surface and both primary and secondary fibres thicker than in *Psammocinia alba* sp. n.; *P. perforodosa* Cook & Bergquist, 1998, a massive, compact sponge without conules, with a folded surface (800 µm thick) armoured by sand, foreign spicules and rocky fragments; *P. maorimotu* Cook & Bergquist, 1998, a lobate sponge with oscula on top, a surface with grooves and ridges and primary fibres with a thickness of 349 µm. From South Korea and China, the following species have been described: *P. conulosa* Lee & Sim, 2004, a massive sponge with ectosomal membrane covered by sand but devoid of circular meshes, oscula scattered and sharp conules 2–4 mm high; *P. ulleungensis* Lee & Sim, 2004, dark grey in colour, with a smooth surface and thick, slightly fasciculated, primary fibres (100–300 µm); *P. mammiformis* Sim, 1998, a massive, grey or purple coloured sponge, covered with mammiform protuberances and with very thick choanosomal fibres 550–900 µm; *P. mosulpia* Sim, 1998 mainly differs from *P. alba* sp. n. for its crust of sand and foreign spicules not organised in circular meshes; *P. jejuensis* Sim, 1998, characterised by tick fibres (up to 470 µm) and by filaments with large terminal knobs (12–20 µm in diameter); *P. gageoensis* Sim & Lee, 2001, has no detritus in the fasciculated primary fibres. Both *P. samyangensis* Sim & Lee, 1998 and *P. wandoensis* Sim & Lee, 1998 differ from *P. alba* sp. n. mainly in the thickness of the secondary fibres. Finally, *P. rubra* Sim & Lee, 2002 differs from *P. alba* sp. n. for its red colour and the larger size (up to 320 µm) and colour (reddish-brown) of the fibres.

The other species of *Psammocinia* have a particular morphology, very different respect to *Psammocinia alba* sp. n.; *P. arenosa* (Lendenfeld, 1888) and *P. hawere* Cook & Bergquist, 1996 are cup-shaped sponges. *Psammocinia halmiformis* (Lendenfeld, 1888) is irregularly lamellate and *P. vesiculifera* (Poléjaeff, 1884) is a tube sponge. *Psammocinia amodes* Cook & Bergquist, 1998 is a spatulate sponge with a thin, semi-cylindrical basal portion for anchoring to the substrate, while *P. bergquistae* Sim & Lee, 2001 has a thumb shape and secondary fibres, forming a secondary web.

Due to the difficulties to differentiate, in some cases, species of the genus *Psammocinia* from other taxa of the family Irciniidae, we also examined the species belonging to *Ircinia* and *Sarcotragus* from the Indo-Pacific area. All these species are different from *Psammocinia alba* sp. n. in morphology, fibre thickness, and structure (see below).

The incorporation of foreign material can play several roles in sponge growth. Usually, this behaviour is explained just as strengthening of the sponge tissue, but other roles could be considered, e.g. the enhancement of sponging fibre production (Cerrano et al. 2007).

####### Genus *Ircinia* Nardo, 1833

######## Ircinia colossa

Taxon classificationAnimaliaDictyoceratidaIrciniidae

Calcinai, Bastari, Bertolino & Pansini sp. n.

http://zoobank.org/3547C559-C615-420B-874F-6782568B7D40

Figure 10

######### Material examined.

Holotype: MSNG 60141, PH-44, 15/01/2005, Timur (Bunaken Island), about 20 m depth. Paratype: MSNG 60142, BKA 25, 12/09/2014, Yellow coco (Bangka Island), about 20–25 m depth.

######### Other material.

BU-590, 27/07/2004, Timur (Bunaken Island), 25 m depth. INDO-431, 13/05/2005, Jetty (Siladen), depth not stated, N01°37'38.8"; E124°48'00.8".

######### Diagnosis.

Soft and elastic cup-shaped *Ircinia* with a large, central cavity; conulose surface; heavily fasciculated fibres with foreign material.

######### Description.

**Figure 10.**
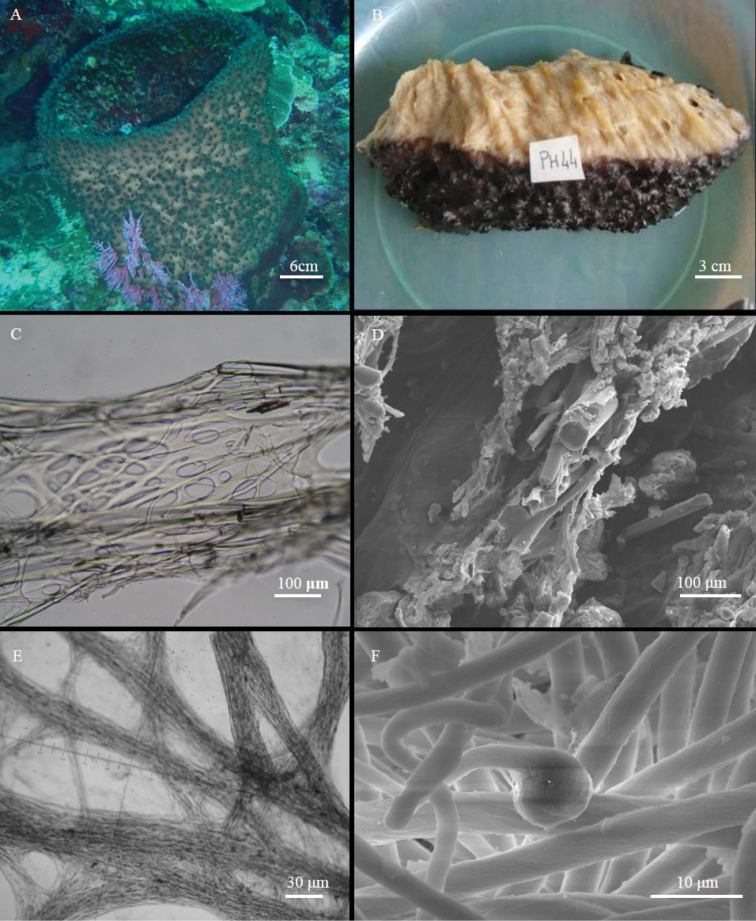
*Ircinia colossa* sp. n. **A** specimen BU-590 *in situ* **B** portion of the holotype **C** fasciculated fibres **D** primary fibres with foreign spicules **E** filaments organised in tracts **F** filaments with a terminal knob in evidence.

The sponge is columnar, reminding of a partially hollow cylinder, due to the presence of a wide central cavity (Fig. 10A). It may be as high as 80 cm, with a wall 1–2 cm thick. The holotype is a fragment approximately 4.5 × 2 cm. The external colour is light brown with greenish tinges on the conules and on the rim of the cavity (Fig. 10A). The freshly collected sponge is beige inside (Fig. 10B). Alcohol-preserved specimens remain almost the same in colour. The sponge surface is strongly conulose, with rounded or slightly flattened conules 2–4 mm high (Fig. 10A, B). The oscula (3–5 mm in diameter) are present in the inner part of the central cavity. Consistence is soft and elastic, but the sponge is difficult to tear off.

**Skeleton.** The choanosomal skeleton is formed by primary fibres cored by foreign spicules (Fig. 10C, D), 180–350 µm in diameter and heavily fasciculated (Fig. 10C). They are connected by secondary fibres 50–80 µm in diameter, sometimes cored by single spicules. The fibres form a reticulation of elongated meshes, 100–150 µm in size, and cribrose plates (Fig. 10C). Very abundant thin filaments are mainly organised in tracts (Fig. 10E), but also dispersed in the mesohyl. They are 3-5 µm thick and present an oval or rounded terminal knob (7.5–10 µm in diameter) (Fig. 10F).

######### Etymology.

The name refers to the sturdy and large size of the sponge.

######### Remarks.

The studied specimens are attributed, according to Cook and Bergquist 2002, to the genus *Ircinia* for the strong fasciculation of fibres, with foreign material inside and the presence of filaments. There are more than 40 species of massive, encrusting, digitate or branching *Ircinia* in the Indo-Pacific area (van Soest et al. 2016), which differ from *Ircinia colossa* sp. n. in morphology, fibre thickness and quantity of external debris in the skeleton.

Only two species of *Ircinia*, living between 10 and 40 m depth in the temperate water of South-East Australia, show a central cavity: *I. caliculata* (Lendenfeld, 1888) and *I. rubra* (Lendenfeld, 1889). *Ircinia caliculata* differs from *I. colossa* sp. n. in the general morphology, colour, and organisation of the fibres. It has the rim of the cup bent outwards; the internal part of the cavity with small conules 2–3 mm high. The external part of the sponge presents digitate processes about 10 mm thick. The colour is dark-red brownish. It has fasciculated fibres full of sand grains. *Ircinia rubra* differs from *I. colossa* sp. n. in the general shape and fibre size. It is a small, conical, pedunculate sponge with a central cavity. All the fibres are full of debris and foreign spicules and the secondary fibres, 100 µm in diameter, are thicker than those of *Ircinia colossa* sp. n.

We also examined species belonging to the genus *Sarcotragus*; none of them fits with the characters of the new species. *Sarcotragus aliger* (Burton, 1928) is clavate, cylindrical with an apical osculum and fibres 80 µm in diameter, while *S. australis* (Lendenfeld, 1888) is a massive red sponge. *Sarcotragus coreanus* (Sim & Lee, 2002) is massive to encrusting, beige in colour; *S. gapaensis* Sim & Lee, 2000 is subspherical, dark brown to black, with big primary fibres 280–530 µm in diameter. *Sarcotragus maraensis* Sim & Lee, 2000 is globular with sharp conules 2–8 mm high and an ivory and purple colour. *Sarcotragus myrobalanus* (Lamarck, 1814) is an ovoid sponge with a long peduncle, brown-reddish in colour; *S. tuberculatus* (Poléjaeff, 1884) has fibres that often do not ramify and its surface, greyish in colour, is covered by rounded tubercles; filaments are roundish and 55 µm in diameter.

*Ircinia colossa* sp. n. is frequent in the Bunaken Park and the nearby Bangka Island (North Sulawesi); the paratype was found with other relatively large specimens (50 cm high or more) near a hot vent flowing from a sandy bottom (Bertolino et al. 2017).

This species is probably present also throughout northern Australia and Papua New Guinea (J. Hooper, pers. comm.). Molecular analysis, compared against sequences made by Pöppe et al. (2011) for *Ircinia* and *Psammocinia* species from northern Australia, would be very useful to confirm if *Ircinia colossa* sp. n. and *P. alba* sp. n. are also present in Australia.

## Conclusions

The marine diversity in Indonesia is still far from being well known. The present contribution highlights the underexplored diversity of Porifera in this area, suggesting the presence of a very high number of still undescribed species. Thanks to this impressive diversity, the areas here considered are important spots for diving tourism, requiring the urgent development of sustainable tourism practices. In particular, at Bangka Island, mining activities are rapidly damaging reef integrity, even if this process is currently strongly counteracted by the local population. It is worth noting that also there, as in many other strongly populated areas, the conflict between the need to preserve local biodiversity and the economic development can quickly lead to a lose-lose equilibrium.

Generally, the economic value of biodiversity is still far from being adequately understood; in particular, the actual value of sponges in the maintenance of the homeostasis of a reef needs to be studied in more detail.

In temperate regions affected by climatic anomalies, filter feeders are among the most affected functional categories (Coma et al. 2009, Di Camillo and Cerrano 2015), and negative trends of sponge diversity and abundance have been reported from several areas (Wulff 2013).

The area of the present study is very rich in terms of diversity, but the baseline needs urgent implementation and constant update to avoid the possibility of disregarding changes.

We have documented 94 sponge species from three small spots of the northern tip of Sulawesi. Since 1989, van Soest has reported approximately 830 species from Indonesia; the species recorded here represent only a small part of the astonishing sponge diversity of the area.

The coral triangle is known for its high level of biodiversity and continuously, in recent years, new marine organisms have been described. Moreover, many authors (see for example Barber et al. 2000) have demonstrated strong regional genetic differentiation even across short distances and even for reef organisms presumed to be subjected to rapid dispersion even between distant populations. Sponge diversity across Indonesian coral reefs could be extraordinarily underestimated considering the limited capacity of sponge larval dispersal (Maldonado and Bergquist 2002). Unfortunately, for North Sulawesi and the rest of the archipelago, both collecting and taxonomic efforts remain limited.

The listed taxa (Table 1) sometimes include well-known sponge species because reef sponges of Indonesia are also present in the Indo-Pacific area (van Soest 1990); for other poorly known species, specimen photos and short taxonomic notes may assist for further identification, supporting future, desirable monitoring work (Supplementary files 1 and 2).

## Supplementary Material

XML Treatment for Aaptos lobata

XML Treatment for Tethytimea tylota

XML Treatment for Stelletta tethytimeata

XML Treatment for Rhabdastrella distincta

XML Treatment for Amphimedon cf. sulcata

XML Treatment for Amphimedon anastomosa

XML Treatment for Niphates laminaris

XML Treatment for Psammocinia alba

XML Treatment for Ircinia colossa

## Supplementary materials

Supplementary material 1 Underwater photos of the species. Data type: species data This dataset is made available under the Open Database License (http://opendatacommons.org/licenses/odbl/1.0/). The Open Database License (ODbL) is a license agreement intended to allow users to freely share, modify, and use this Dataset while maintaining this same freedom for others, provided that the original source and author(s) are credited.Barbara Calcinai, Azzurra Bastari, Giorgio Bavestrello, Marco Bertolino, Santiago Bueno Horcajadas, Maurizio Pansini, Daisy M. Makapedua, Carlo Cerrano

Supplementary material 2 Additional remarks of the species Data type: species data This dataset is made available under the Open Database License (http://opendatacommons.org/licenses/odbl/1.0/). The Open Database License (ODbL) is a license agreement intended to allow users to freely share, modify, and use this Dataset while maintaining this same freedom for others, provided that the original source and author(s) are credited.Barbara Calcinai, Azzurra Bastari, Giorgio Bavestrello, Marco Bertolino, Santiago Bueno Horcajadas, Maurizio Pansini, Daisy M. Makapedua, Carlo Cerrano
