# Supplementary figures and images for: Demosponge diversity from North Sulawesi, with the description of six new species
Source: Zookeys. 2017 Jun 20;(680):105–50. doi: 10.3897/zookeys.680.12135 (PMC5523382; doi:10.3897/zookeys.680.12135)

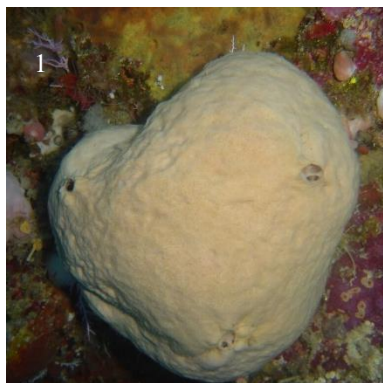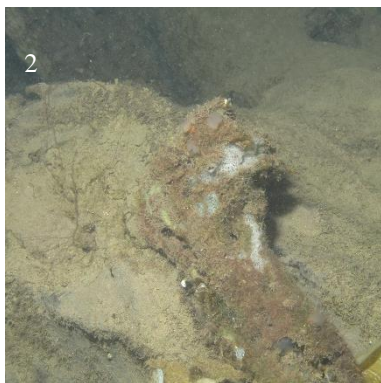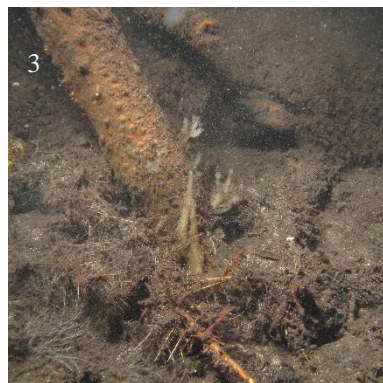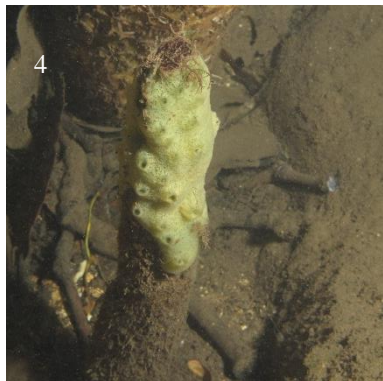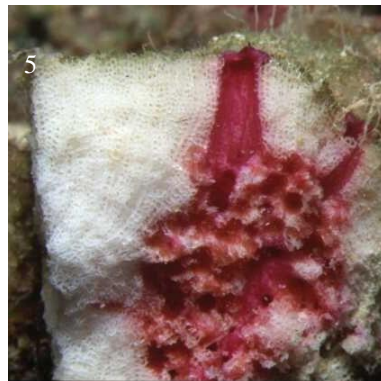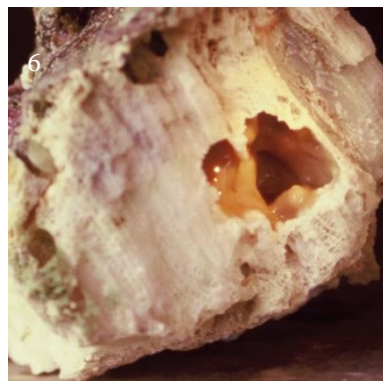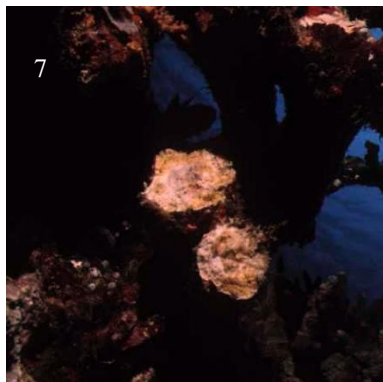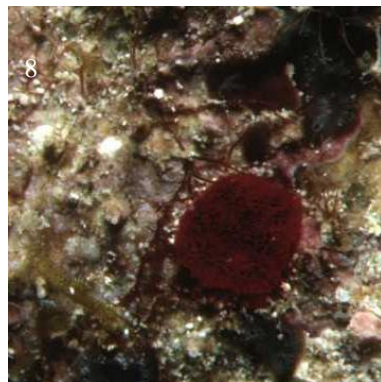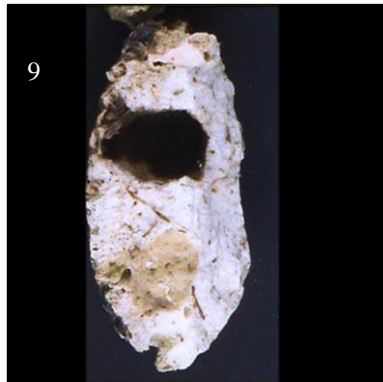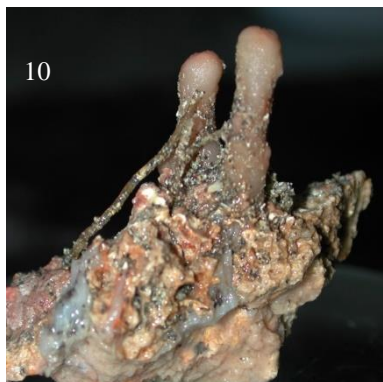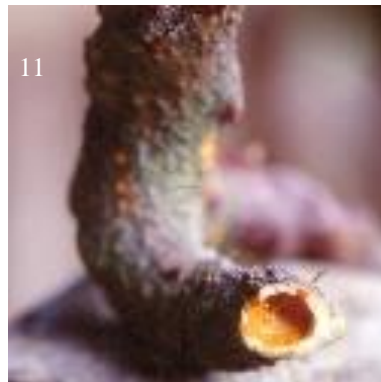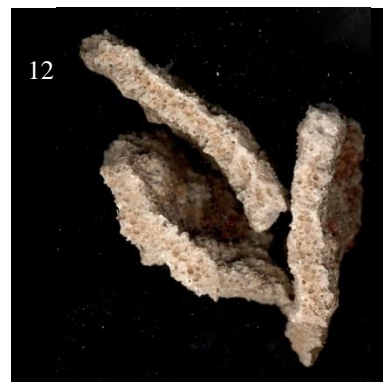

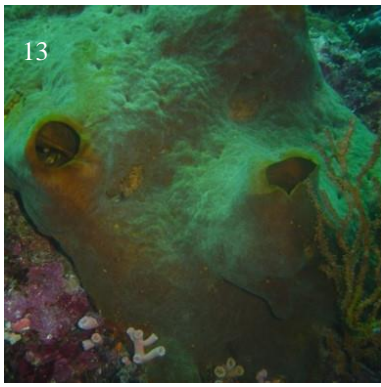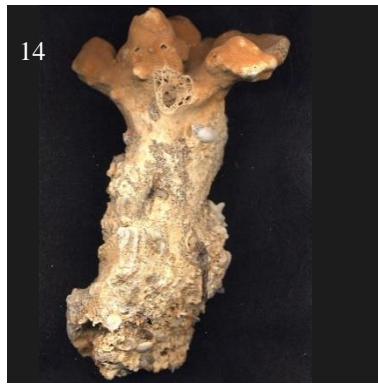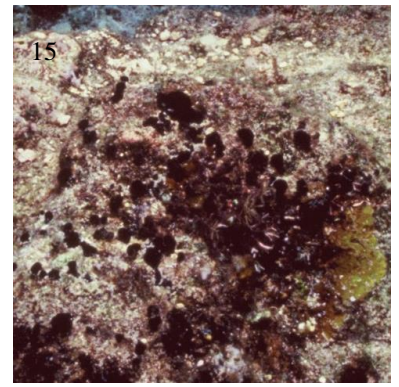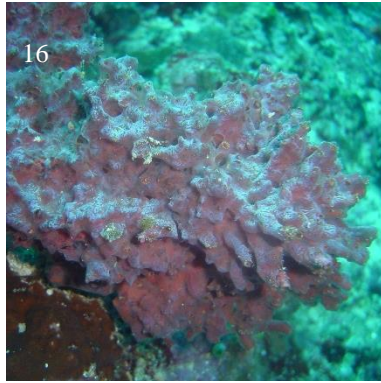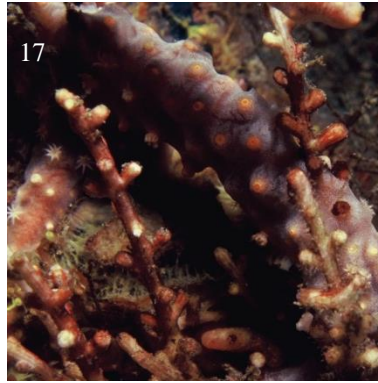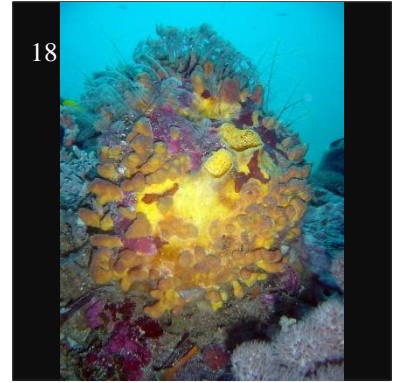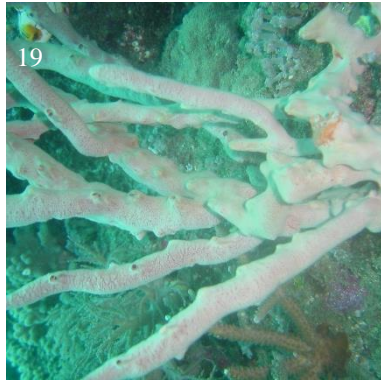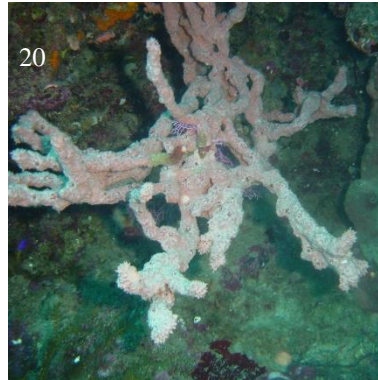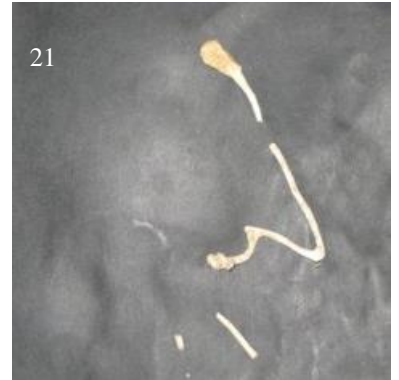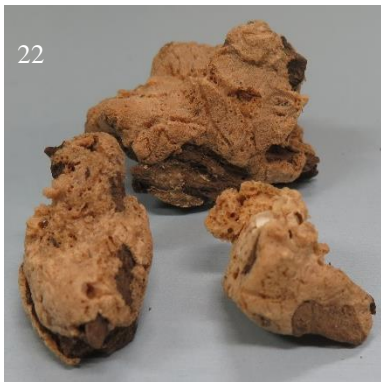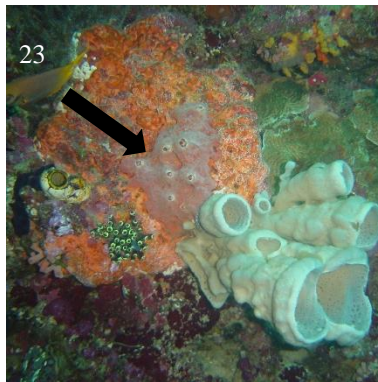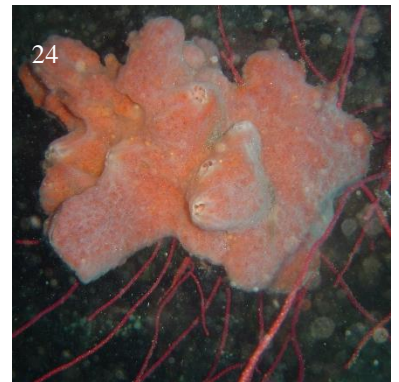

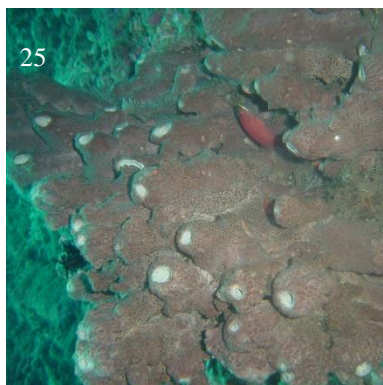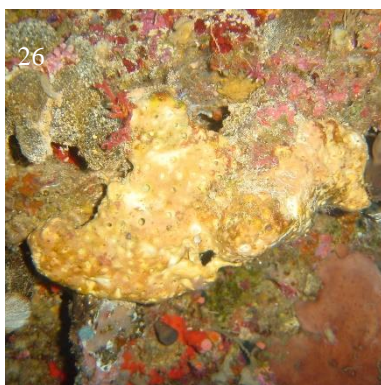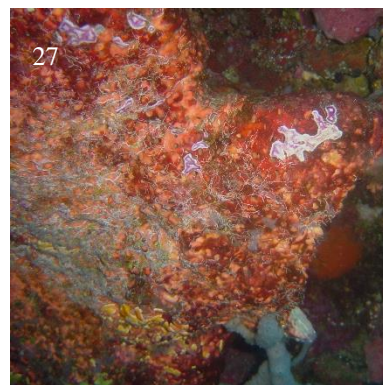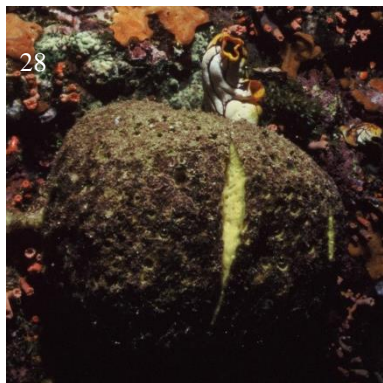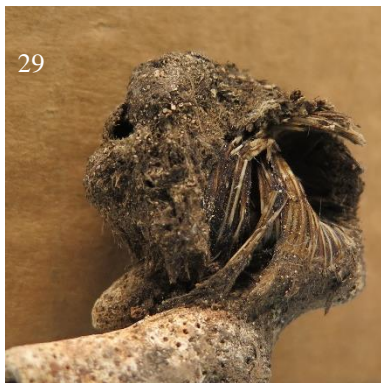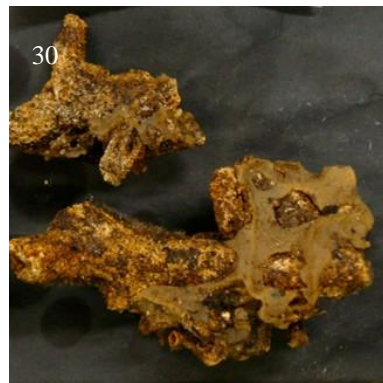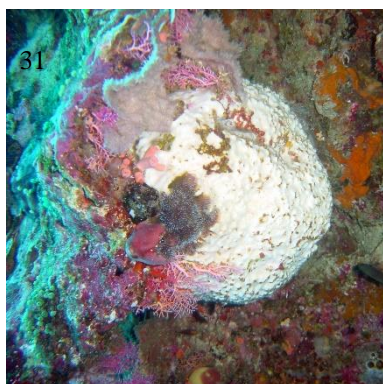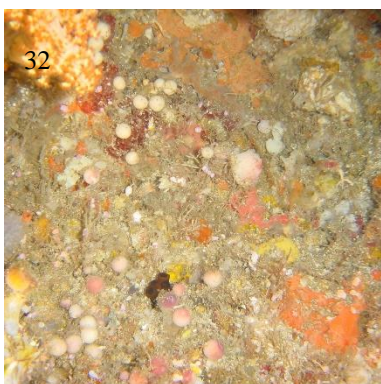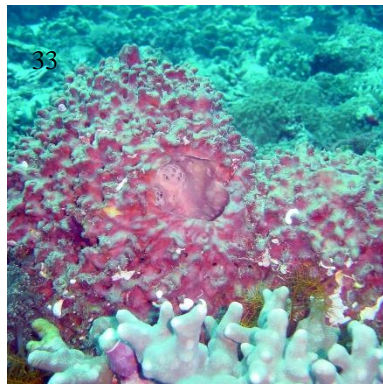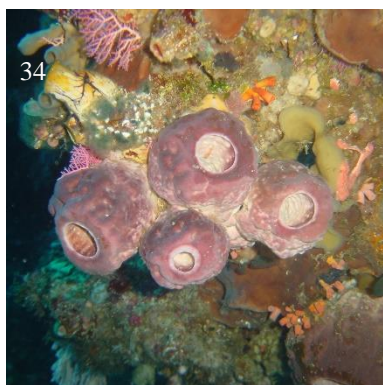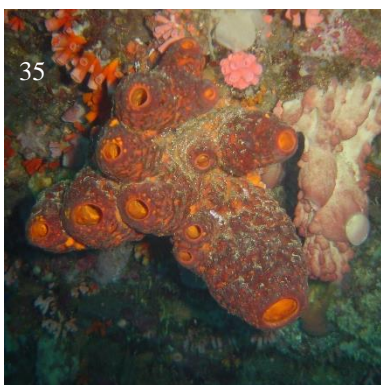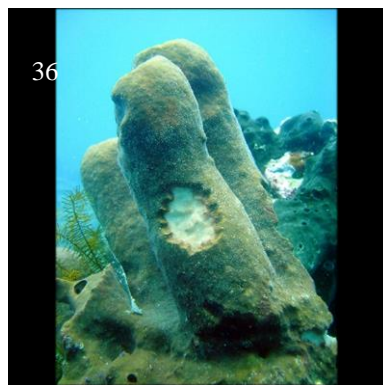

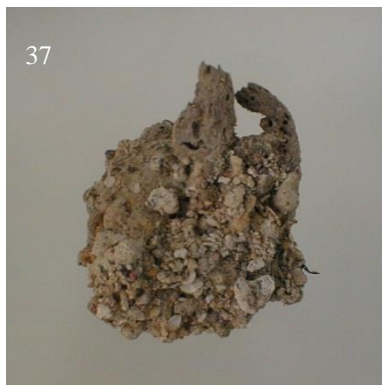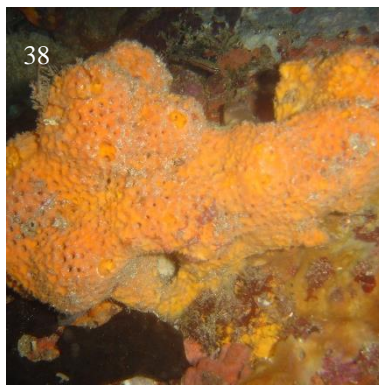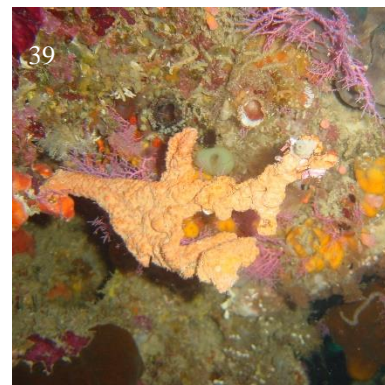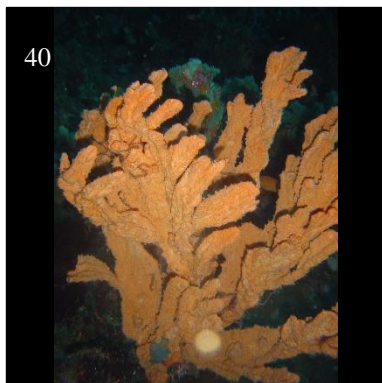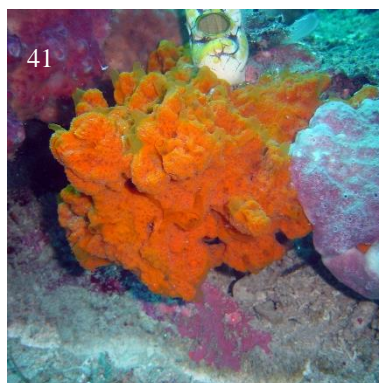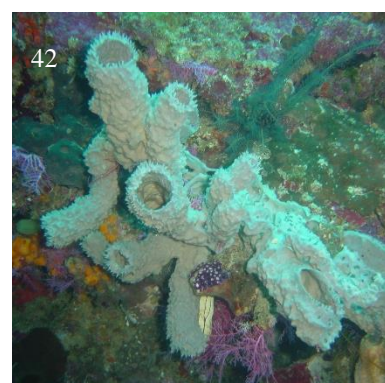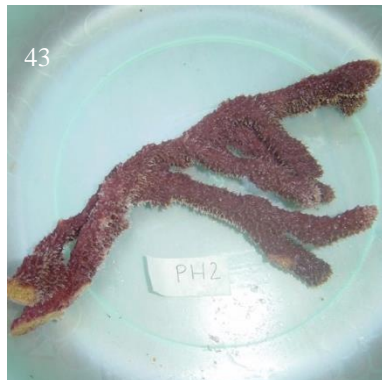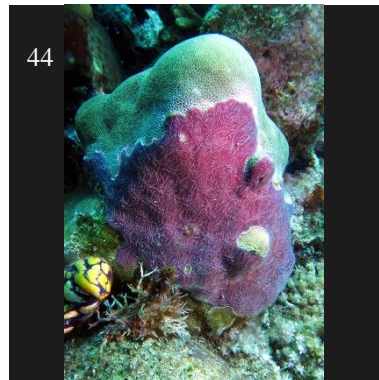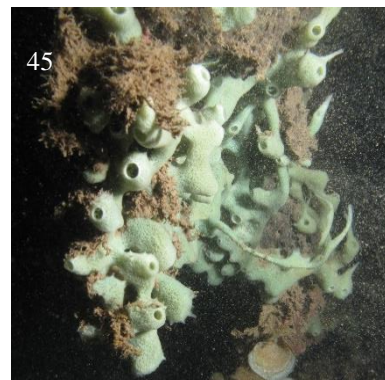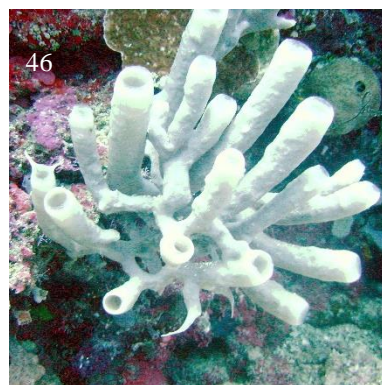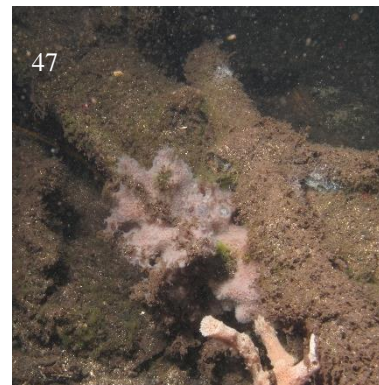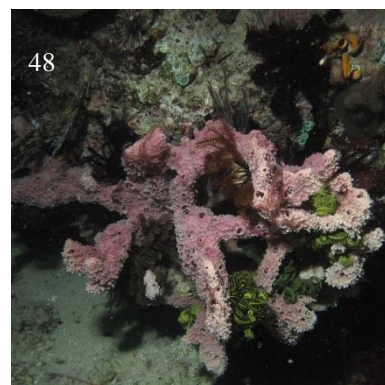

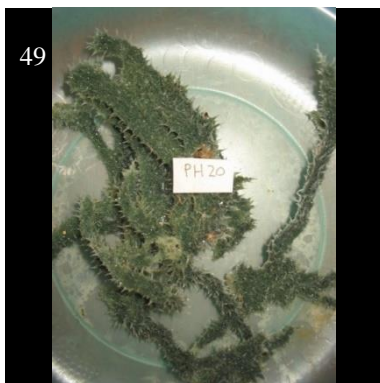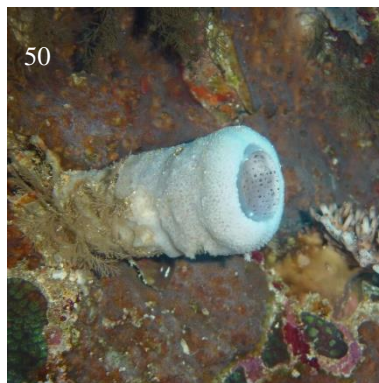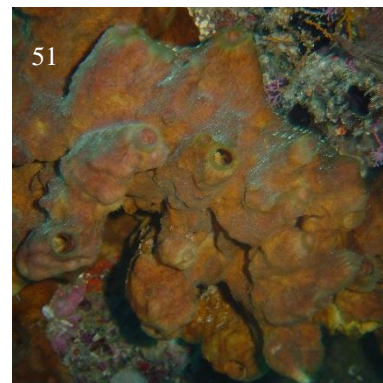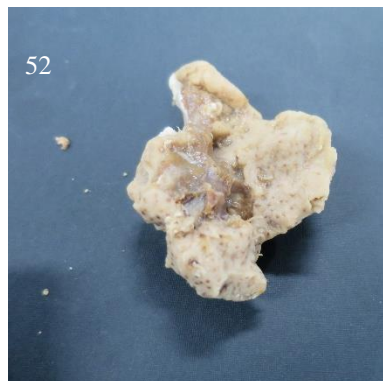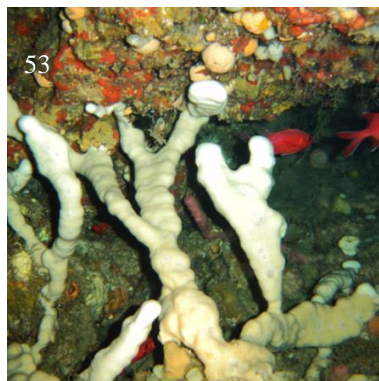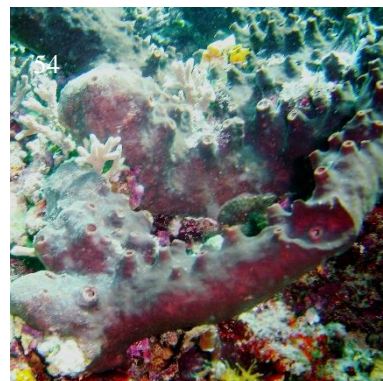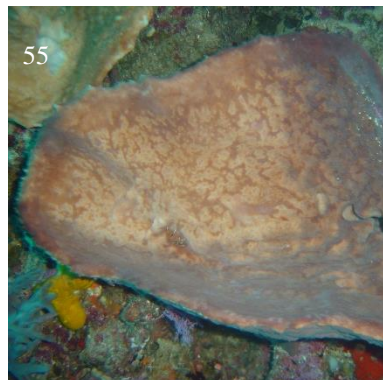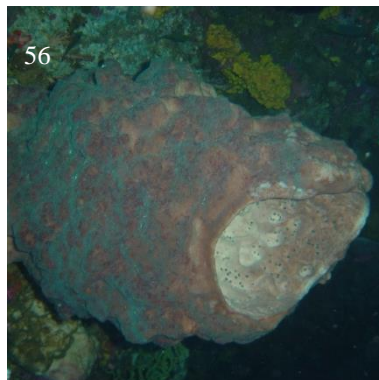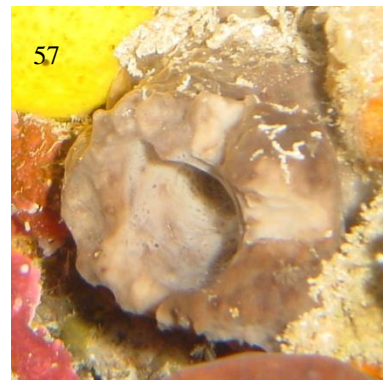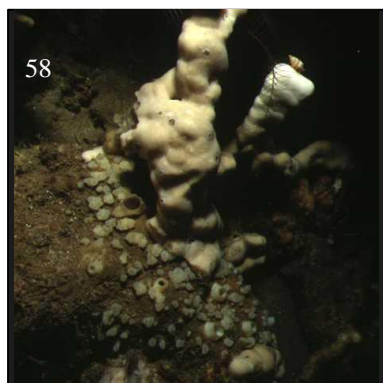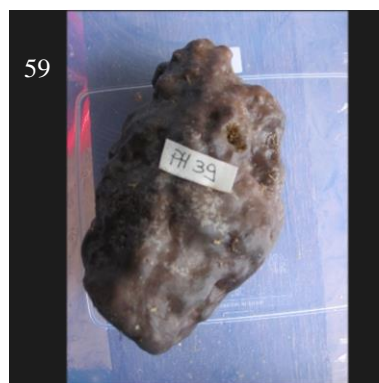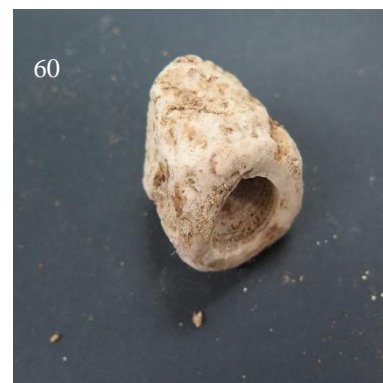

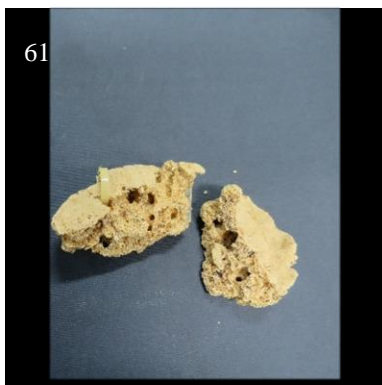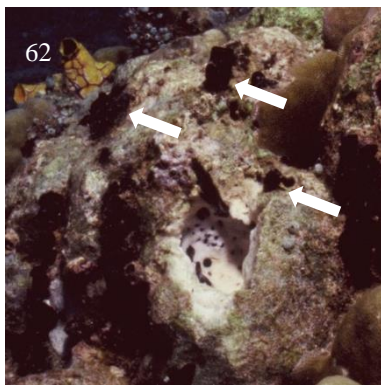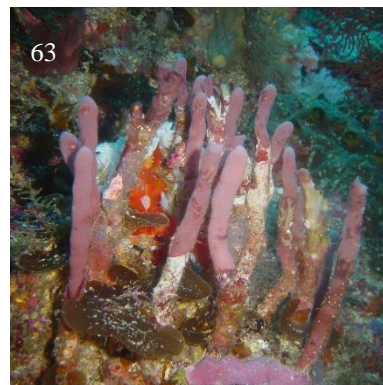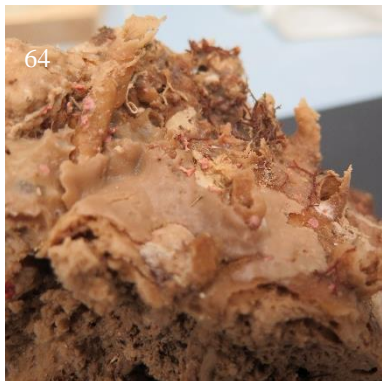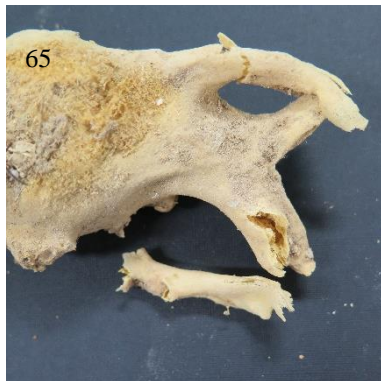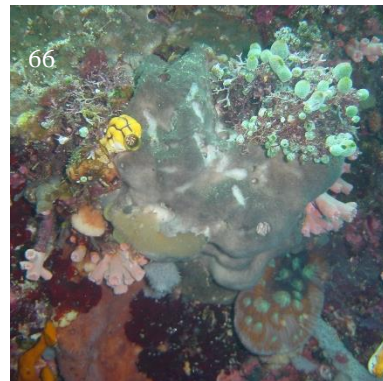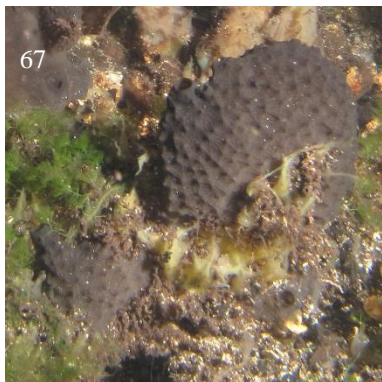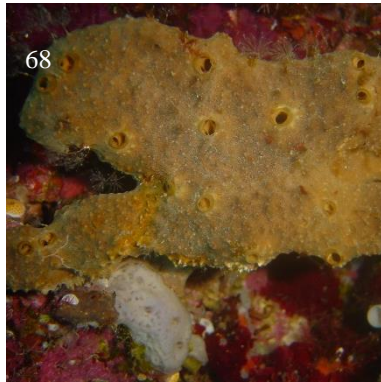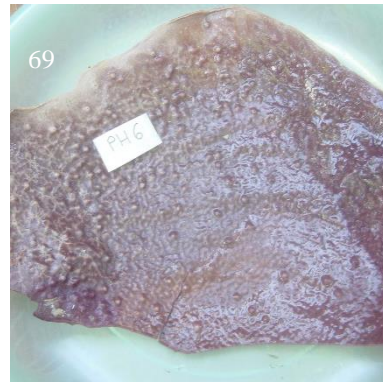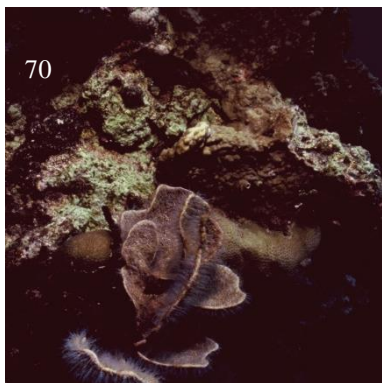

Supplement: Supplementary material 1 — Underwater photos of the species. [file zookeys-680-105-s001.pdf]
