# Supplementary material for: Demosponge diversity from North Sulawesi, with the description of six new species
Source: Zookeys. 2017 Jun 20;(680):105–50. doi: 10.3897/zookeys.680.12135 (PMC5523382; doi:10.3897/zookeys.680.12135)

SUPPLEMENTARY FILE 2

***Spirastrella pachyspira* Lévi, 1958**
Figure 12
The species was found inside a small cavity, about 70 mm^2 wide, of a dead coral. Tylostyles are straight with a well-formed, rounded head, and acerate or sometimes blunt tips (Fig. 12A); they measure 210-(535.1±179.3)-920 x 5-(20.8±7)-32.5 µm. Four types of microscleres: large, straight spirasters with branched rays and flat, spiny tips 77.5-(96.5±13.7)-125 x 7.5-(11.2±3.3)-15 µm (Fig. 12B, C); short spirasters, often amphiaster-like, with curved axis 7.5-(12±2.4)-15 x 2-3 µm (Fig. 12D); stout spirasters with spiralled axis and long, conical spines 32.5-(81.4±16.6)-105 x 5-(8.1±2.9)-12.5 µm (Fig. 12E); some thinner forms are also common; slender spirasters, straight or bent, with few spines 37-(66.6±13.8)-85 x 2.5-(3.7±1.1)-5 µm.

The specimen fits with the species described by Lévi (1958) in the presence of four categories of spirasters encompassing the large spirasters with flat tips.

This species is known from the Red Sea and the Indian Ocean.

***Mycale* (*Mycale*) *corallina* Calcinai, Cerrano and Bavestrello, 2016**
Synonyms: *Mycale* (*Mycale*) *vansoesti* Calcinai, Cerrano, Totti, Romagnoli & Bavestrello, 2006, (Non: *Mycale* (*Grapelia*) *vansoesti* Hajdu, 1995).

M. vansoesti sensu Calcinai, Cerrano, Totti, Romagnoli and Bavestrello, 2006 is a junior homology of *Mycale* (*Grapelia*) *vansoesti* Hajdu, 1995. This homology is here emended following articles 52, 57 and 60 of the International Code of Zoological Nomenclature (http://www.nhm.ac.uk/hosted-sites/iczn/code/) and the *nomen novum* is here proposed (articles 67 and 72 of ICZN).
Etymology: after the association with a living coralline alga.

Following the ICZN (articles 72 and 75), a neotype is not designated and the *nomen novum (M. (M.) corallina)* and the earlier name *Mycale* (*Mycale*) *vansoesti* Calcinai, Cerrano, Totti, Romagnoli and Bavestrello, 2006 are synonyms and have the same name-bearing type: MSNG 52665; Paratype: MSNG 52803; Type locality: Siladen Island, North Sulawesi.

***Podospongia colini* Sim-Smith and Kelly, 2011**
Figures 11(21), 13
A small specimen in dried state; collected in 1998 in the area around Bunaken Park; no additional data available. It is a small, ovoid, peduncolate sponge with one oscular opening at the top; the stalk is about 8-9 cm long and the body 1 x 0.5 cm in diameter. The basal part of the stalk is slightly enlarged to assure the anchorage. The colour is whitish-cream and the consistency is quite hard. In the stalk, anisostrongyles are organised in intermingled tracts; in the body, the strongyloxeas are grouped in tracts radially disposed. Spicules are very thin strongyloxeas in two categories 530-(700.7±105.6)-880 x 5-(7.9±2.4)-10 and 290-(394±73.2)-490 x 5-(6.7±1.2)-7.5 µm (Fig. 13A); anisostrongyles 380-(455±36.9)-520 x 10-(14.2±1.6)-15 µm (Fig. 13B); numerous aciculospinorhabds (Fig. 13C) in various stages of spinosity and development (protospinorhabds) (Fig. 13D); protospinorhabds sigmoid (Fig. 13E-F)) 7.5-(46.8±21.6)-87.5 x 2.5-(6.7±3.5)-12.5 µm.

The sponge fits, for general morphology, skeletal organisation, size and shape of spicules, with the species described by Sim-Smith and Kelly in 2011.

It was known only from the type locality (Tarabitan, northern tip of Sulawesi).

***Tedania* (*Tedania*) *coralliophila* Thiele, 1903**
Figure 11(23)
Encrusting sponge growing on a specimen of *Agelas nakamurai* Hoshino, 1985. The colour in life is pinkish, orange with white rims around the elevated oscula. Surface is smooth and consistency quite hard in dried state. In the ectosome, the tylotes are tangentially arranged, while in the choanosome,

there is a plumoreticulate skeleton of styles. Styles are slightly curved 240-(262±10.8)-270 x 6.2-(7.9±1.2)-10 μm; tylotes are straight, with spined and slightly enlarged apices 235-(262.6±10.3)-280 x 3.7-(4.5±0.5)-5 μm; rhaphides 275-(307±19.7)-350 x about 2 μm.

***Stelletta clavosa* Ridley, 1884**
Figure 11(32)
This species, very common in the Marine Park of Bunaken, is always associated to polychetes living inside the sponge. Brief descriptions of the association are to be found in Calcinai et al. (2005b) and Cerrano et al. (2006).

***Theonella mirabilis* (de Laubenfels, 1954)**
Figures 11(35), 14
Massive, vase sponge made of coalescent tubes with oscules on top of the tubes. The colour is brownish, dark orange externally, orange internally. The dried sample is beige, light beige internally. The surface is irregular with small ridges or protuberances. The consistency is hard when dried. In the ectosome, spinose rhabds and tangential strongyles create a continuous layer.
No phyllotriaenes. In the choanosome, strongyles are arranged in tracts creating a messy reticulation; desmas are dispersed and more concentrated in the deeper part of the sponge.

Strongyles straight or slightly curved 440-(514±36.1)-560-μm (Fig. 14A); desmas very characteristic, with smooth clads except at the extremities, where they branch and develop groups of conical spines (Fig. 14B); clads are 80-(102±11.31)-115 μm (N=15); microrhabds rounded or pointed at the ends (Fig. 14C, D) and covered with minute spines 7.5-(9.5±1.5)-12.5 μm.

***Thoosa letellieri* Topsent, 1891**
Figure 15
The species is characterised by nodulose amphiasters typical of *Thoosa*, with numerous intermediate forms 12.5-(16.7±1.5)-1.5 μm (Fig 15A, B); amphiasters with eight thin rays ending with group of spines 25-(28.2±2.7)-32.5 μm (Fig. 15B); oxeas straight, with spines at the extremities 127.5-(143.9±8.9)-157.5 μm (Fig. 15A); toxas 75-(88.3±8.5)-μm (Fig. 15A); thin raphides, curved in the middle 25-(28.2±32.5)-μm (Fig. 15A).
Van Soest et al. 2016 consider the status of this species as "*taxon inquirendum*" and report its distribution for the Celtic Sea. Topsent (1881, page 581) describes this species as boring into a *Tridacna* shell. According to the geographical range of these bivalves (restricted to the Indo-Pacific region), the distribution of the associated boring species needs to be considered for the Indo-Pacific.

The specimen found in Indonesia excavating into a *Tridacna* fits for its spicular feature and in particular for its spined oxeas and raphides with that described by Topsent.

***Acanthostrongylophora ingens* (Thiele, 1899)**
Figures 11(51), 16
In the Marine Park of Bunaken, this species is frequently associated with polychetes. This association was described in Calcinai et al. (2005b) and Cerrano et al. (2006), which reported a symbiont density inside the sponge (as *Acanthostrongylophora ashmorica* Hooper, 1984) of more than 185 individuals/cm^3.

Specimens from Bunaken are characterised by strongyles (Fig 16. A) very variable in size 49.4-(111.7±22.7)-143 x 2.6-(5.7±1.4)-7.8 μm; thin oxeas 85-108.02(13.05)-137.5 x <2.5 μm (Fig. 16B, C). The examination of *A. ingens* (type material on loan from NMB, n NMB2012/009; holotype n 41) revealed the presence of strongyles 75-(136.11± 23.78)-162.5 x 5-(6.6±1.1)-7.5 μm (Fig. 16D) and of thin oxeas 112.5-(128.7±10.5)-140 x <2.5 (Fig. 16E) not described by Thiele (1899). Another species of *Acanthostrongylophora, A. ashmorica,* has been described by Hooper (1984) from Australia and later re-described after holotype examination by Desqueyroux-Faúndez and Valentine (2002), which corroborated the validity of the genus. The species, characterised by

strongyles and thin oxeas, is very close to *A. ingens*. The presence of thin oxeas in the holotype of *A. ingens* supports the idea of van Soest et al. 2016, who consider *A. ashmorica* as a junior synonym of *A. ingens.*

Figure legends
Supplementary files

Figure 11. Photographs of the species collected in the North Sulawesi and listed in table 1. **1** *Plakortis lita*; **2** *Amorphinopsis excavans*; **3** *Ciocalypta tyleri*; **4** *Topsentia halichondrioides*; **5** *Cliona jullieni*; **6** *Cliona mucronata*; **7** *Cliona orientalis*; **8** *Cliona schmidtii*; **9** *Cliona utricularis*; **10** *Cliothosa aurivilli*; **11** *Cliothosa hancocki*; **12** *Pione carpenteri*; **13** *Spheciospongia solida*; **14** *Spheciospongia vagabunda*; **15** *Zyzzya fuliginosa*; **16** *Monanchora enigmatica*; **17** *Desmapsamma vervoorti*; **18** *Coelocarteria agglomerans*; **19** *Clathria* (*Thalysias*) *cervicornis*; **20** *Clathria* (*Thalysias*) *mutabilis*; **21** *Podospongia colini*; **22** *Tedania* (*Tedania*) *brevispiculata*; **23** *Tedania* (*Tedania*) *coralliophila* black arrow; **24** *Tedania* (*Tedania*) *dirhaphis*; **25** *Agelas ceylonica*; **26** *Agelas mauritiana*; **27** *Agelas nakamurai*; **28** *Cinachyrella australiensis*; **29** *Tetilla ridleyi*; **30** *Dercitus* (*Stoeba*) *bangkae*; **31** *Rhabdastrella globostellata*; **32** *Stelletta clavosa*; **33** *Melophlus sarasinorum*; **34** *Theonella cylindrica*; **35** *Theonella mirabilis*; **36** *Theonella swinhoei*; **37** *Biemna fortis*; **38** *Acanthella cavernosa*; **39** *Phakettia ridley*; **40** *Stylissa carteri*; **41** *Stylissa massa*; **42** *Callyspongia* (*Cladochalina*) *aerizusa*; **43** *Callyspongia* (*Cladochalina*) *fibrosa*; **44** *Chalinula nematifera*; **45** *Cladocroce burapha*; **46** *Haliclona* (*Reniera*) *fascigera*; **47** *Haliclona* (*Halichoclona*) *centrangulata*; **48** *Dasychalina fragilis*; **49** *Gelliodes fibulata*; **50** *Niphates olemda*; **51** *Acanthostrongylophora ingens*; **52** *Neopetrosia seriata*; **53** *Neopetrosia similis*; **54** *Petrosia* (*Petrosia*) *hoeksemai*; **55** *Petrosia* (*Petrosia*) *nigricans*; **56** *Petrosia* (*Petrosia*) *plana*; **57** *Petrosia* (*Petrosia*) *seychellensis*; **58** *Petrosia* (*Strongylophora*) *corticata*; **59** *Petrosia* (*Strongylophora*) *durissima*; **60** *Petrosia* (*Strongylophora*) *strongylata*; **61** *Xestospongia testudinaria*; **62** *Siphonodictyon mucosum* white arrows; **63** *Oceanapia fistulosa*; **64** *Oceanapia seychellensis*; **65** *Oceanapia toxophila*; **66** *Spongia* (*Spongia*) *ceylonensis*; **67** *Hyrtios communis*; **68** *Hyrtios reticulatus*; **69** *Phyllospongia papyracea*; **70** *Carteriospongia foliascens*.

Figure 12. *Spirastrella pachyspira* Lévi, 1958 **A** tylostyles **B, C** large spirasters with flattened, spiny tips **D** short spirasters **E** stout spiraster with elongated axis.

Figure 13. *Podospongia colini* Sim-Smith and Kelly, 2011 **A** small strongyloxeas **B** anisostrongyles **C** aciculospinorhabds **D** protospinorhabd **E, F** sigmoid protospinorhabds.

Figure 14. *Theonella mirabilis* (de Laubenfels, 1954) **A** strongyle with a magnification of a strongyle tip **B** Desma **C** rounded microrhabds **D** pointed microrhabd.

Figure 15. *Thoosa letellieri* Topsent, 1891 **A** Oxea (white arrow), toxa (black arrow), thin raphide (empty arrow) and nodulose amphiasters **B** nodulose amphiaster (black arrow) and amphiaster with eight thin rays (white arrow).

Figure 16. *Acanthostrongylophora ingens* (Thiele, 1899) **A** Strongyles **B** thin oxeas **C** magnification of the extremity of an oxea **D** strongyles and thin oxea in **E** of the holotype (NMB2012/009; holotype n 41).

Figure 12

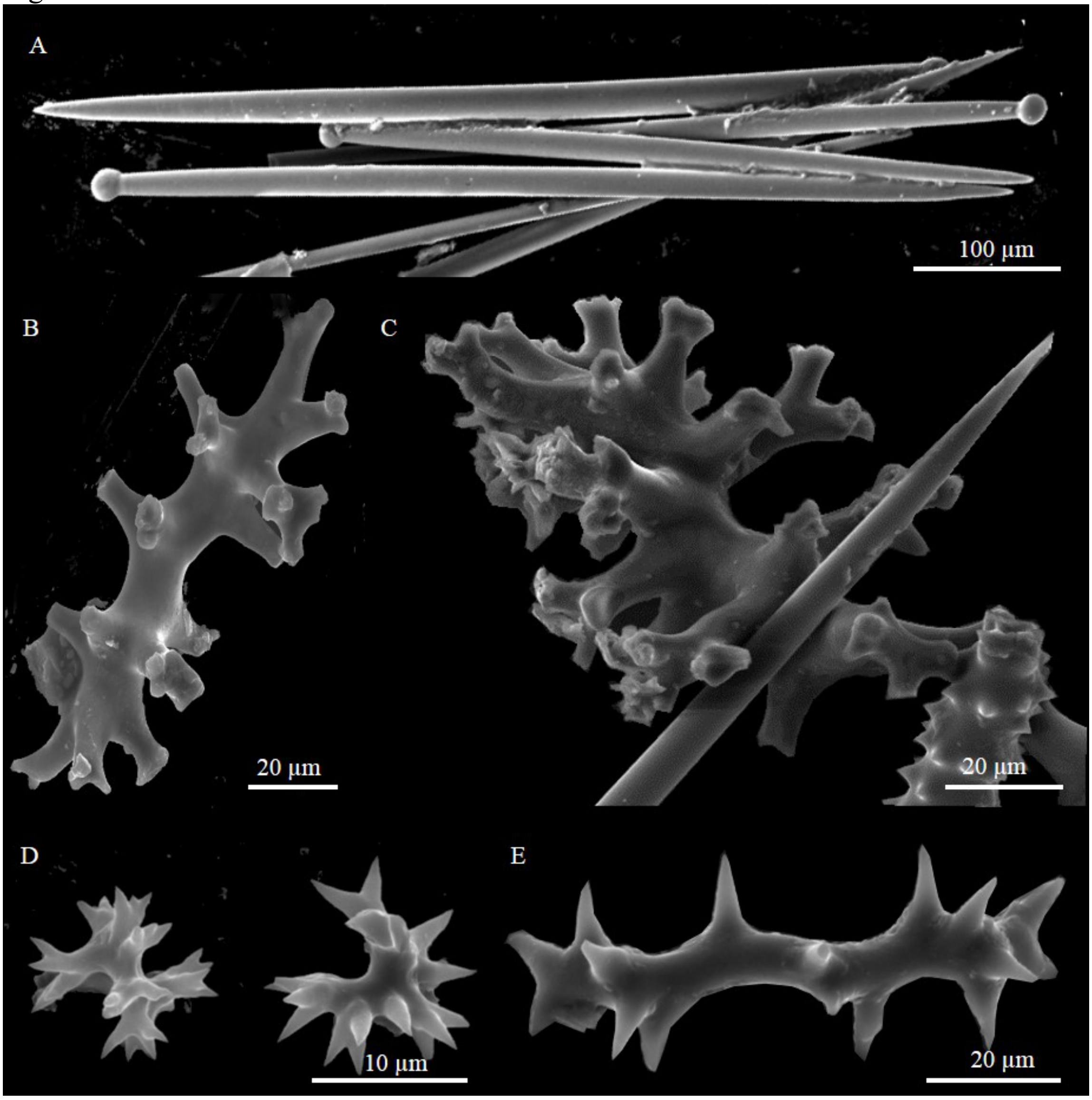

Figure 13

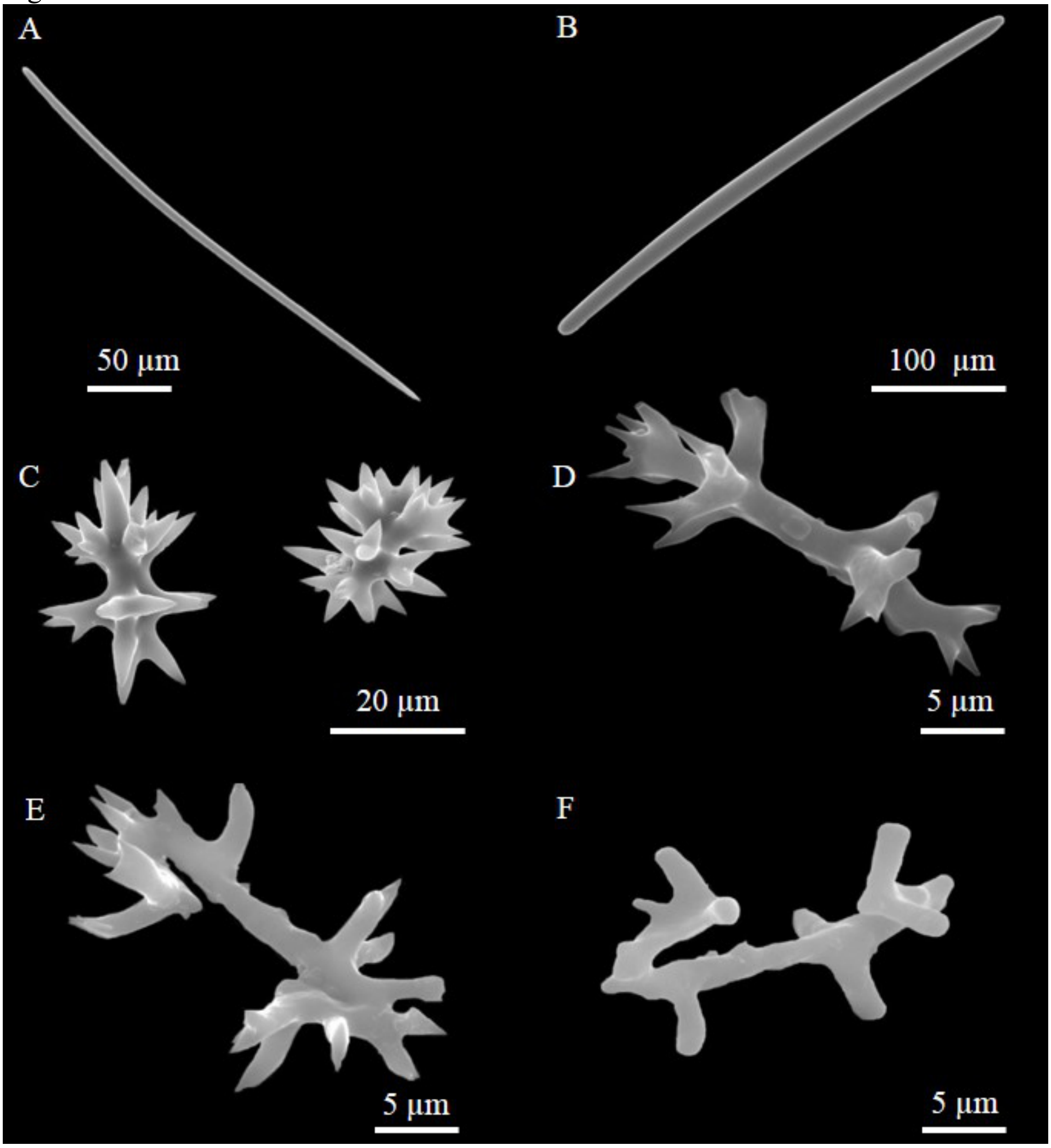

Figure 14

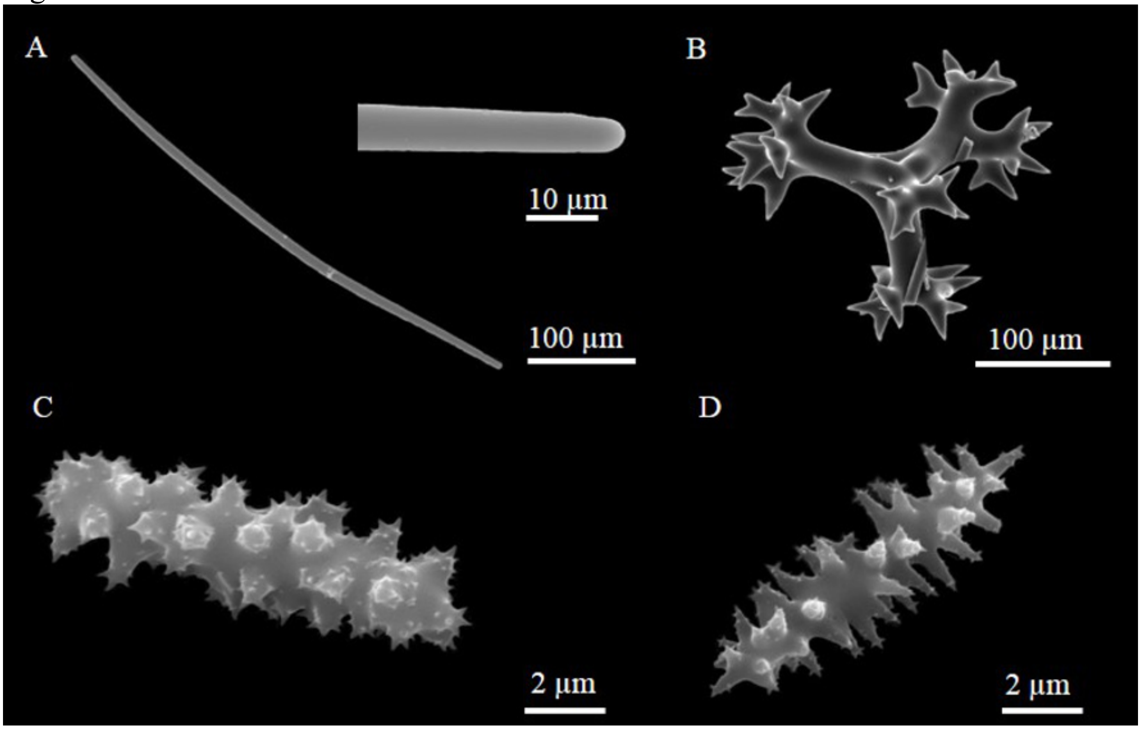

Figure 15

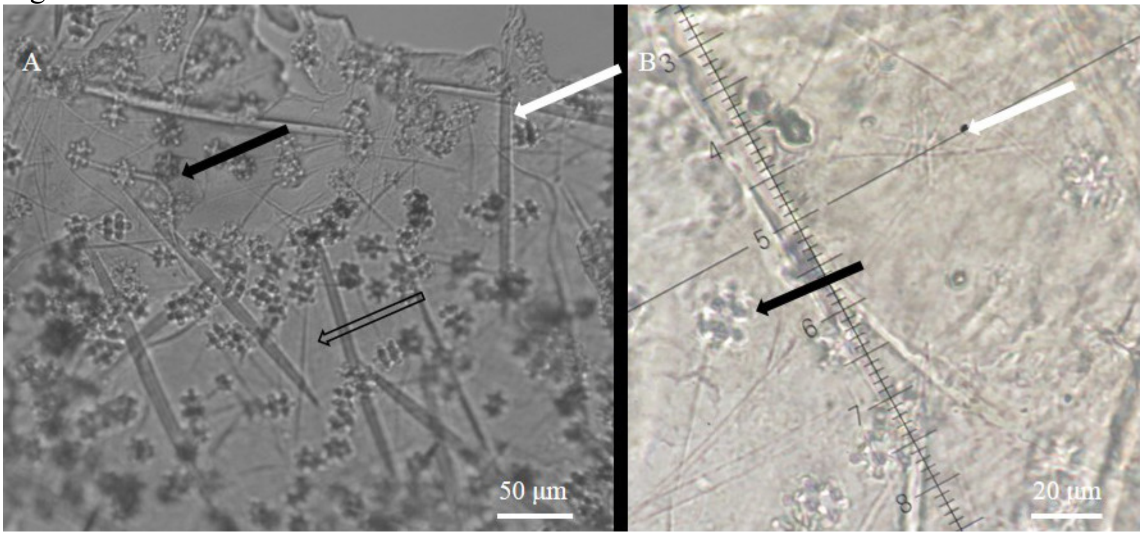

Figure 16

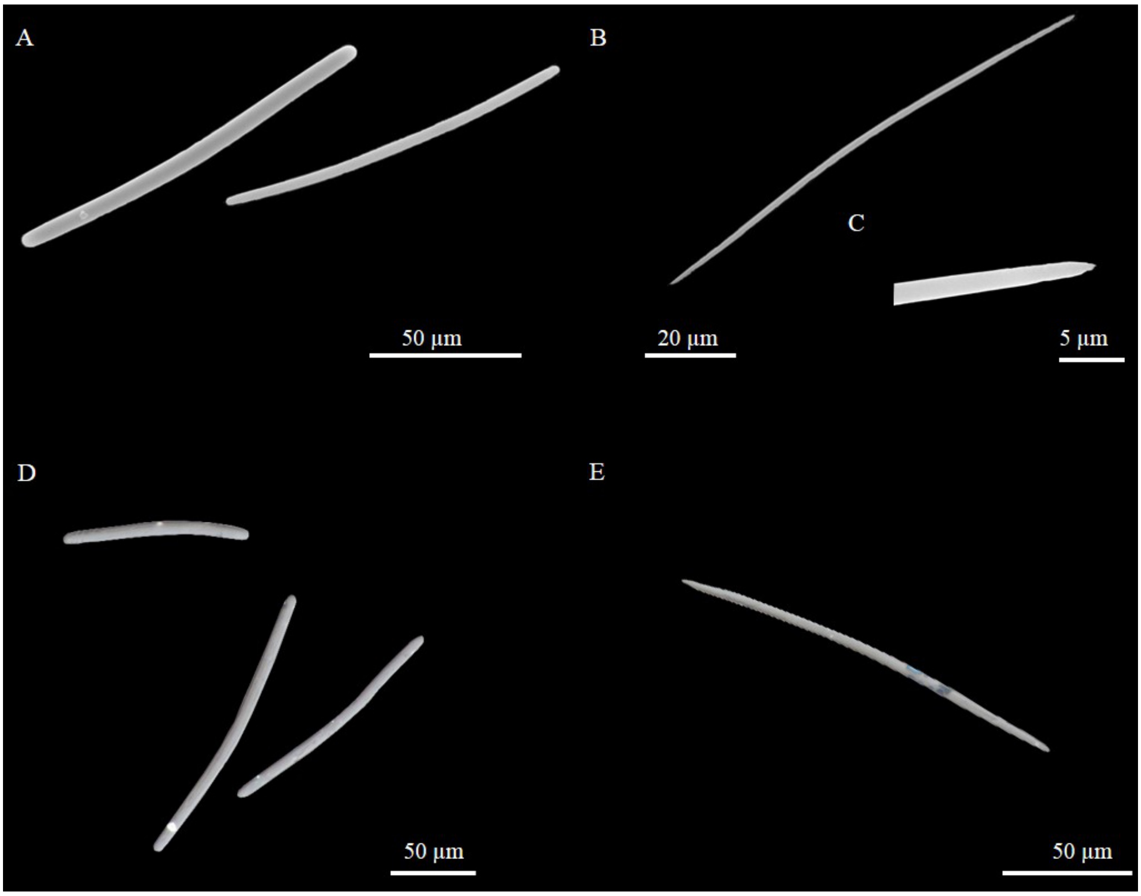

Supplement: Supplementary material 2 — Additional remarks of the species [file zookeys-680-105-s002.pdf]
